# Plant diversity influences plant volatile emission with varying effects at the species and community levels

**DOI:** 10.1073/pnas.2518326123

**Published:** 2026-01-15

**Authors:** Pamela Medina-van Berkum, Cynthia Albracht, Maximilian Bröcher, Marcel Dominik Solbach, Gideon Stein, Michael Bonkowski, François Buscot, Anna Heintz-Buschart, Anne Ebeling, Nico Eisenhauer, Tarek S. El-Madany, Yuanyuan Huang, Karl Kuebler, Sebastian T. Meyer, Jonathan Gershenzon, Sybille B. Unsicker

**Affiliations:** ^a^Department of Biochemistry, Max Planck Institute for Chemical Ecology, Jena 07745, Germany; ^b^Plant-Environment-Interactions Group, Botanical Institute, Kiel University, Kiel 24118, Germany; ^c^Biosystems Data Analysis, Swammerdam Institute for Life Sciences, University of Amsterdam, Amsterdam 1000 BE, The Netherlands; ^d^Department Soil Ecology, Helmholtz Centre for Environmental Research, Halle 06120, Germany; ^e^Institute for Biosafety in Plant Biotechnology, Julius Kühn Institute, Quedlinburg 06484, Germany; ^f^Population Ecology Group, Institute of Biodiversity, Ecology and Evolution, University of Jena, Jena 07743, Germany; ^g^Terrestrial Ecology Group, Institute of Zoology, University of Cologne, Cologne 50674, Germany; ^h^Computer Vision Group, Faculty of Mathematics and Computer Science, University of Jena, Jena 07743, Germany; ^i^Experimental Interaction Ecology, German Centre for Integrative Biodiversity Research, Halle-Jena-Leipzig 04103, Germany; ^j^Institute of Biology, Leipzig University, Leipzig 04103, Germany; ^k^Field experiments and instrumentation, Max Planck Institute for Biogeochemistry, Jena 07445, Germany; ^l^Terrestrial Ecology Research Group, Department of Ecology and Ecosystem Management, School of Life Sciences Weihenstephan, Technical University of Munich, Munich 85350, Germany

**Keywords:** Biodiversity and ecosystem functioning (BEF), chemodiversity, herbivory, plant–plant communication, *Plantago lanceolata*

## Abstract

Studies have investigated the interactions between plants through competition and resource sharing to understand the mechanisms behind the positive effects of plant diversity on productivity. Volatile organic compounds (VOCs) are important info-chemicals in plant–plant interactions, but they have so far rarely been considered in this context. Here, we measured VOC emissions at the community scale and for one species (*Plantago lanceolata*) in experimental plant communities of varying diversity (The Jena Experiment) to understand the role of VOCs in driving biodiversity-ecosystem functioning relationships. We show that plant diversity determines the release of plant VOCs at both scales. At the community level, plant species richness directly enhanced VOC emission and increased VOC richness both directly and indirectly by altering leaf area index. At the species level, plant diversity did not directly affect the VOC emissions of *P. lanceolata* but indirectly affected it by influencing the VOC emissions from the surrounding community. *P. lanceolata* individuals in communities with high concentrations of green leaf volatiles decreased their VOC emission, while those in communities with high concentrations of terpenoids increased their VOC diversity. Our results provide evidence that plant diversity shapes community-level plant VOC emission and thus influences focal plant VOC emission inside the community.

## Significance

Plant volatiles are key info-chemicals mediating plant–environment interactions, but their role in biodiversity–ecosystem functioning relationships remains unknown. Using an experimental grassland, we studied how plant diversity affects volatile release at both the community level and the species level. We demonstrate that with increasing plant diversity, the amount and diversity of volatiles released by the community increase. While volatile profiles of the focal species *Plantago lanceolata* did not directly respond to plant diversity, they were indirectly influenced by the surrounding community emissions. Our findings show that plant diversity shapes community-level volatile emissions and, in turn, alters the release of volatiles from individual plants, revealing a route through which biodiversity can affect ecosystem functioning.

Understanding how plant diversity affects community structure and interactions has been a major driver of research in biodiversity and ecosystem functioning (BEF) relationships in recent years. Field experiments have shown that plant diversity not only increases plant community productivity but also causes changes in both primary and specialized metabolites in grassland plant species (1–3). As a result, individuals of the same species growing in environments with varying diversity may exhibit differences in their trait expression, such as in their metabolite profiles, as they experience differing abiotic and biotic pressures (4–6).

Plants emit a large variety of volatile organic compounds (VOCs), including green leaf volatiles, terpenoids, and aromatics (7). These metabolites are released by all types of plant organs in response to both biotic and abiotic stimuli (8) and they fulfill a plethora of functions. For instance, VOCs can act as signals for pollinators, parasitoids, or mycorrhizal fungi, as defense compounds to repel antagonists like herbivorous insects and pathogens (9–11) and as important signals in intra- and interspecific plant communication (12, 13). By mediating these interactions, VOCs might enhance the plant adaptability to a variety of environmental challenges (14) and thus provide indicators of changes in their abiotic and biotic environment.

The variety of VOCs arises from the participation of several different core pathways and possibly additional enzymes (7). VOC diversity can thus vary in terms of the number of compounds, their relative abundance, and their biosynthetic origins. Although plants emit VOCs constitutively, the high diversity of their emitted blends also arises from responses to other organisms and abiotic environmental factors (14). Numerous studies have shown that herbivore or pathogen attacks drastically change plant VOC profiles quantitatively and qualitatively (15–17). Moreover, environmental factors, such as drought and light limitation, have also been recognized to influence the VOC profiles of the plants (18). Consequently, the complexity of the surrounding plant community, moderated by plant diversity, might shape their VOC emissions.

An increase in plant diversity within a community leads to changes in both abiotic and biotic interactions (19, 20). This complexity raises the question of how VOC emissions respond to variation in plant diversity. While most VOC studies have focused on the plant species level (5, 21), the effects of plant diversity on VOC emissions can occur at different scales, with factors influencing patterns at the species level differing from those at the community level. At the species level, leaf damage can directly influence VOC profiles, but it can also have indirect effects. Exposure to herbivore- or pathogen-induced plant volatiles, for example, can trigger changes in the VOC profiles of nearby receiving plants and consequently reduce subsequent herbivore damage (22–24). Notably, these responses can vary depending on the neighboring community and their identity (21, 25). Previous research found that *Trifolium pratense* L. altered its VOC diversity in response to both intra- and interspecific competition, suggesting that community diversity influences VOC-mediated herbivory responses (21). Similarly, *Plantago lanceolata* L. showed a decreased VOC diversity with increasing plant diversity (5), further supporting the role of community composition in shaping VOC profiles.

At the plant community level, research on VOC emission has largely been theoretical, with limited experimental evidence. Plant diversity could directly influence VOC complexity through the increase of plant species richness or plant phylogenetic diversity (26). However, it may also have indirect effects on VOC profiles by modifying below- and above-ground abiotic and biotic factors. For example, increasing plant species diversity can increase herbivore load and reduce soil pathogens (27, 28), and both herbivores and soil pathogens are known to influence plant VOC emissions (14). Additionally, increased species diversity can affect abiotic conditions such as light availability, soil temperature, and moisture (29). An increase in leaf area index (LAI) or vegetation height can reduce light availability, thereby affecting the emission of light-dependent compounds such as terpenoids (30). These hypotheses offer alternatives to how increasing plant diversity could directly and indirectly influence VOC emissions at the community level, which ultimately may affect emissions at the species level as well.

Despite their ecological importance, most of the research on VOCs has been limited to controlled environments in laboratories or greenhouses. Experts have long called for more studies under natural field conditions (14, 26, 31), but only a limited number of research projects have explored the VOC profiles of plants under natural ecosystems (31–33). Furthermore, most of these studies have focused on individual plants and species, with few examples at the community level (34). Studies of VOC emission by communities with varying numbers of species are especially lacking. Since changes in VOC profiles, both at the species and community levels, may have a major influence on ecosystem functioning by altering interactions with other organisms, it is important to better understand the factors affecting VOC emission under field conditions in complex plant communities.

In this study, conducted in the framework of a long-term grassland biodiversity experiment [The Jena Experiment (35)], we investigated how VOC profiles vary across an experimental plant diversity gradient at both the community and the species levels, focusing specifically on *P. lanceolata* L. We selected this species due to the available insights into its metabolomic profiles, ecological interactions with other organisms, responses to plant diversity, and sufficient replication across the plant diversity gradient (5, 36, 37). Here, we show that community-level VOC diversity increases with increasing plant diversity, whereas at the species-level, plant diversity does not directly influence the VOC emissions of *P. lanceolata* but rather affects them indirectly by shaping the VOC emissions from the surrounding community. Our results provide insight into the various factors shaping VOC diversity across a plant diversity gradient, highlighting the distinctions between species- and community-level VOC patterns.

## Results

### Community VOC Emission and Richness Increase With Increasing Plant Species Richness.

First, we investigated whether the initially sown gradient in plant species richness, which ranges from monocultures to 60-species mixtures, is still present in the communities under study. In total, 82 plant species belonging to 61 genera, 19 families, and 14 orders were identified in the communities. Twenty-three of these species colonized the communities from the regional species (not belonging to the original species pool of the Jena Experiment, *SI Appendix*, Fig. S1 and Table S1). Realized plant species richness (Hill q0), taxonomic diversity (Hill q1), and phylogenetic diversity (Hill q0 and q1) in the communities were positively correlated with the originally sown plant richness (taxonomic richness: R^2^ = 0.45, *P =* 0.011; taxonomic Shannon diversity: R^2^ = 0.30, *P =* 0.013; phylogenetic richness: R^2^ = 0.35, *P =* 0.005, phylogenetic Shannon diversity: R^2^ = 0.35, *P =* 0.002; *SI Appendix*, Fig. S2).

**Fig. 1.**
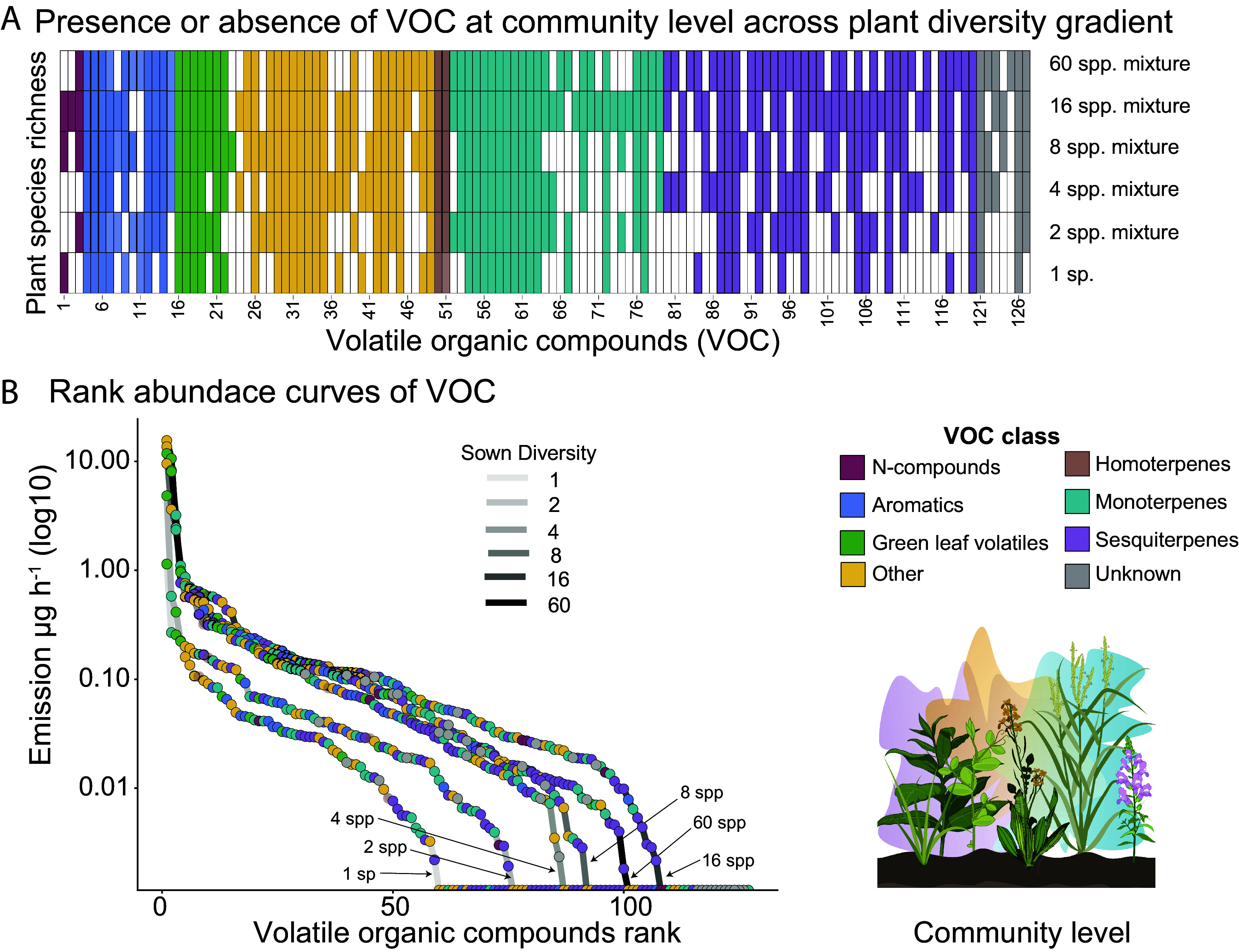
Volatile organic compound (VOC) profiles at the community level across the plant diversity gradient. Headspace VOC emission of experimental grassland communities was collected with a push–pull system (*SI Appendix*, Fig. S7) and analyzed by gas chromatography coupled to mass spectrometry (GC-MS) and flame ionization detector (GC-FID). (*A*) Presence (colored cell) or absence (white cell) of individual VOCs across the plant diversity gradient from monocultures to 60-species mixture plant communities. Each column corresponds to a specific VOC compound, with compound names provided in *SI Appendix*, Table S2. (*B*) Rank-abundance curves of VOCs across the experimental plant diversity gradient. Each dot (color-coded by compound class) represents a VOC. Emission (µg h^−1^) was used as abundance.

We identified 127 VOCs in communities across the gradient in plant species richness. These VOCs were classified into GLVs (green leaf volatiles), fatty acid derivatives other than GLVs, aromatics, nitrogen-containing compounds, homoterpenes, monoterpenes, sesquiterpenes, and a few ungrouped compounds (Fig. 1*A*). Sesquiterpenes and monoterpenes were most numerous, with 41 and 28 compounds, respectively (Fig. 1*A* and *SI Appendix*, Table S2). Furthermore, (*Z*)-3-hexenyl acetate, undecane, and (*E*)-β-ocimene were highly dominant across communities, each representing between 10 to 20% of the VOC profiles on average. In contrast, 41% of the identified compounds contributed less than 0.1% (*SI Appendix*, Fig. S3).

**Fig. 2.**
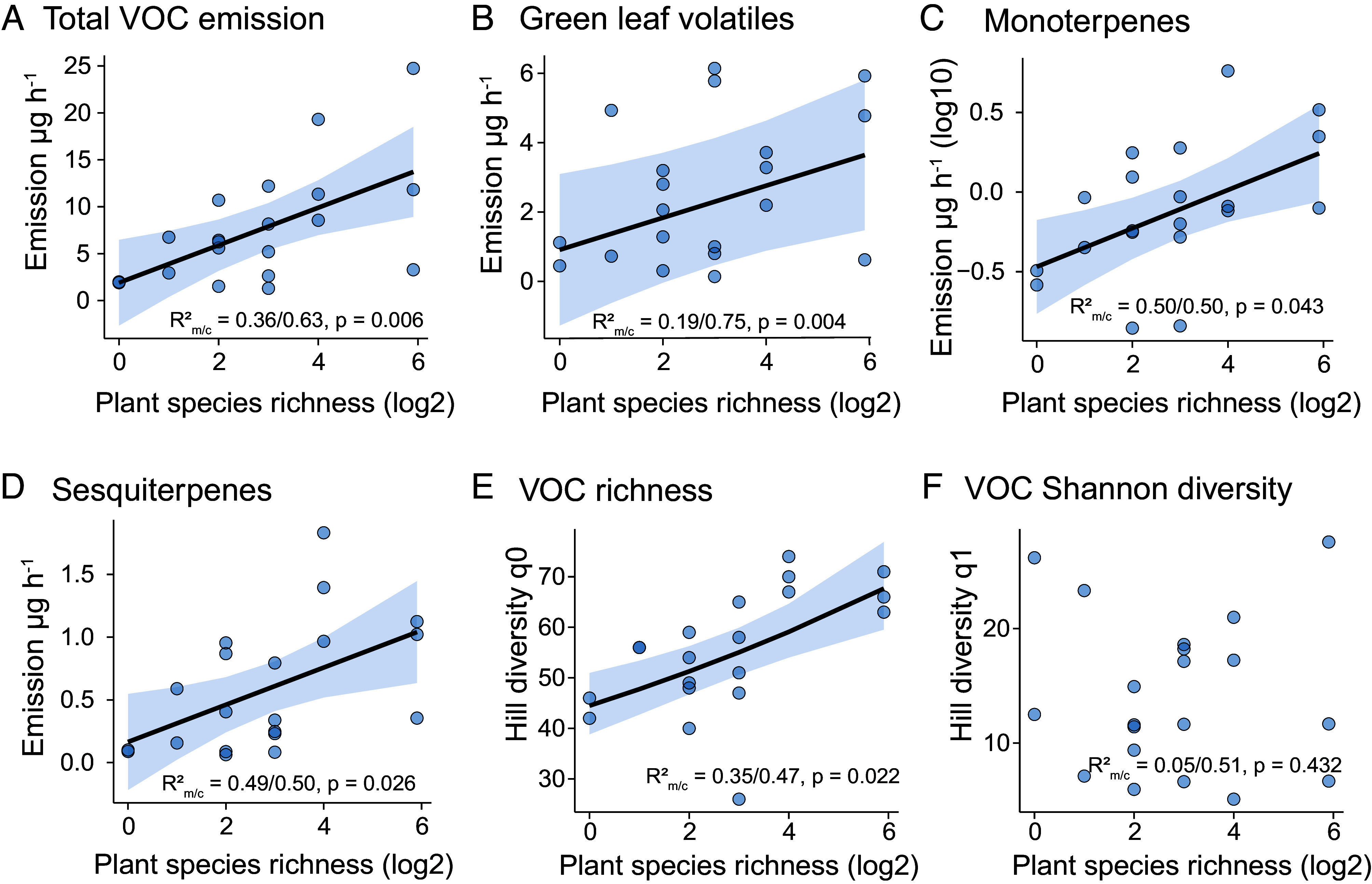
VOC emission and diversity at the community level across the plant diversity gradient. Headspace VOC emission of experimental grassland communities across a plant diversity gradient ranging from monocultures to 60-species mixtures. (*A*–*D*) VOC emission (µg per hour) at the community level, including (*A*) total volatiles, (*B*) green leaf volatiles, (*C*) monoterpenes, and (*D*) sesquiterpenes. (*E*–*G*) VOC diversity at the community level, including (*E*) richness (Hill q0, number of compounds), and (*F*) Shannon diversity (Hill q1) across the diversity gradient. Sample size = 20 communities, with each data point representing the sum of VOC profiles from three random spots within each community. The lines show significant relationships (*P* < 0.05).

Since all plants have species-specific VOCs, we predicted that increasing plant species richness would lead to an increase in the diversity of VOCs emitted from the communities. Indeed, our results show that VOC emission (amount) and richness (number of compounds) increased with increasing plant species richness (emission: *x*^2^ = 7.50, *P =* 0.006; richness: *x*^2^ = 5.26, *P =* 0.022; Fig. 2 *A* and *E*). Terpenoids were most important for the increase in VOC emission (monoterpenes: *x*^2^ = 4.10, *P =* 0.043, sesquiterpenes: *x*^2^ = 4.93, *P =* 0.026, Fig. 2 *C* and *D*), and the increase in VOC richness was mainly driven by sesquiterpenes (*x*^2^ = 7.46, *P =* 0.006, *SI Appendix*, Tables S3 and S4). VOC α-diversity (Hill q1 and Hill q2) did not increase with plant species richness (Hill q1: *x*^2^ = 0.62, *P =* 0.43, Hill q2: *x*^2^ = 1.40, *P =* 0.23; Fig. 2*F*). The rank-abundance curves revealed that the most abundant VOCs are largely consistent across the plant diversity gradient, whereas high-diversity communities exhibited longer tails corresponding to additional low-abundance VOCs (Fig. 1*B* and *SI Appendix*, Fig. S3). These patterns suggest that the addition of species to a community not only contributes new, rarer VOCs but also increases the abundance of dominant compounds. Consequently, these shifts in rare VOCs have limited influence on effective diversity, consistent with the weak response of VOC α-diversity to plant species richness. Despite a strong positive correlation between plant phylogenetic diversity and species richness, VOC α-diversity was not significantly influenced by phylogenetic diversity (*SI Appendix*, Table S3).

Since we observed an increase in the number of VOCs present in community VOC profiles as species richness increases, we quantified compositional dissimilarity using β-diversity metrics to assess changes in VOC profiles across the experimental plant diversity gradient (*SI Appendix*, Fig. S4). We found that VOC β-diversity dissimilarity was highest when low-diversity communities (1- and 2-species mixtures) were compared with more diverse plant communities. This pattern was primarily due to nestedness, meaning that VOCs in low-diversity communities are mostly a subset of those found in more diverse communities. In contrast, differences among communities were smaller when comparisons were made across more diverse plant communities (4- to 60-species mixtures). In these communities, nestedness contributed less to overall β-diversity, whereas VOC turnover was the dominant component explaining compositional differences. This implies a replacement of VOCs, which happens when the addition of new compounds balances out the absence of others. Most of the compounds that were involved in the high turnover proportion were sesquiterpenes (*SI Appendix*, Fig. S4*E*). Notably, plant species β-diversity across the diversity gradient did not mirror the patterns observed for community VOC β-diversity (*SI Appendix*, Fig. S4*A*). This suggests that VOC nestedness and turnover were not directly explained by the plant species β-diversity patterns across communities.

**Fig. 3.**
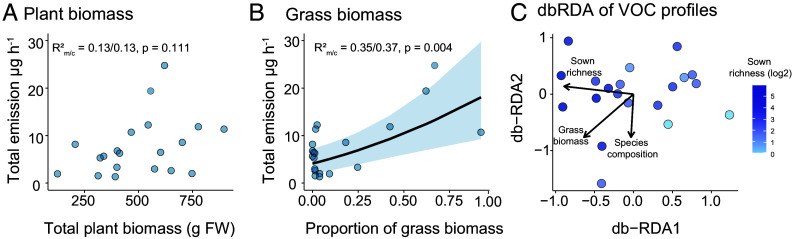
Effect of plant biomass on VOC emission at the community level across the plant diversity gradient. Effect of (*A*) total plant biomass (g FW), and (*B*) proportion of grass biomass on total VOC emission (µg per hour) at the community level. (*C*) db-RDA of community VOC profiles constrained by plant species richness, grass biomass, and plant phylogenetic composition, with collection date as a covariate. Each point represents the sum of VOCs from three random spots per community (sample size = 20 communities). Biomass was measured as the fresh weight of species in the headspace VOC collection cages.

Changes in plant species richness can affect community biomass and thus potentially also impact community VOC emissions. Here, we found neither a significant relationship between community species richness and total plant biomass (*x*^2^ = 2.61, *P* = 0.11), nor between total plant biomass and total VOC emission (*x*^2^ = 2.80, *P* = 0.09; Fig. 3*A*). To account for biomass effects directly, we corrected VOC emissions by total plant biomass (*SI Appendix*, Fig. S5). After this correction, emissions of terpenoids still increased with plant species richness (*SI Appendix*, Fig. S5 *C* and *D*), revealing higher per-unit-biomass emission rates in high-diversity communities. Although total biomass-corrected VOC emission also showed a positive trend with increasing plant species richness, this relationship was not statistically significant, meaning that communities ultimately released comparable overall amounts and numbers of VOCs per unit biomass across the diversity gradient (*SI Appendix*, Fig. S5 *A* and *E*).

Although species richness did not influence plant biomass, plant biomass did correlate positively with the emission of certain compound classes, specifically monoterpenes (*x*^2^ = 9.38, *P* = 0.002) and sesquiterpenes (*x*^2^ = 7.91, *P* = 0.005; *SI Appendix*, Table S3). Notably, while the proportion of legumes and herbs showed no significant effects, the proportion of grass biomass in the community significantly enhanced the total VOC emission (*x*^2^ = 12.28, *P* < 0.001, Fig. 3*B*). Specifically, the presence of grass species like *Bromus erectus* Huds., *Anthoxanthum odoratum* L., and *Dactylis glomerata* L. correlated positively with the overall VOC emission and the emission of some sesquiterpenes (*SI Appendix*, Fig. S6). These results suggest that the composition of plant communities rather than species richness or total biomass alone drives variation in community VOC profiles. Distance-based redundancy analysis (dbRDA) revealed that community-level VOC composition varied along gradients of sown species richness and grass biomass (Fig. 3*C*). According to results from PERMANOVA, both species richness and proportion of grass biomass significantly influenced VOC profiles (sown richness: *F* = 3.07, *P* = 0.003; grass biomass: *F* = 2.15, *P* = 0.012), whereas plant phylogenetic composition did not explain additional variance (*F* = 1.50, *P* = 0.109), even though several individual VOCs were correlated with particular plant species (*SI Appendix*, Fig. S6).

**Fig. 4.**
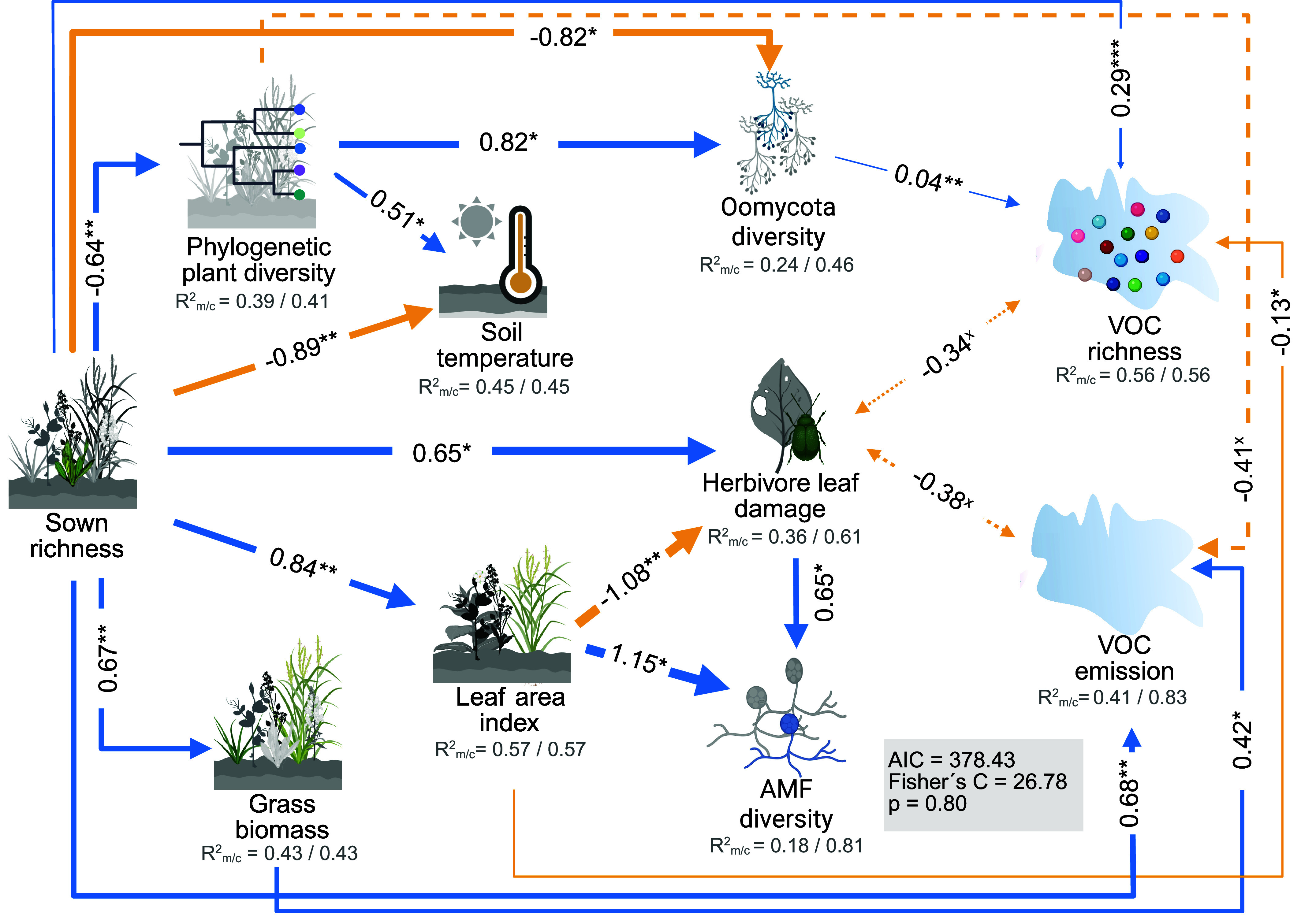
Results from the structural equation model (SEM) investigating the direct and indirect effects of biotic and abiotic conditions on VOC profiles at the community level across a diversity gradient. Blue arrows represent positive effects, while yellow arrows denote negative effects. The width of each arrow is proportional to the strength of the effects, as indicated by the standardized coefficient on each path. Only paths with statistical significance (*P < 0.05, **P < 0.01, ***P < 0.001, solid lines) or a tendency (*×P* < 0.1, dashed lines) are shown. Double-headed arrows represent covariances between variables without an assumed causal direction. Marginal and conditional R^2^ for component models are reported under the response variables (marginal before the slash and conditional after). Sample size = 20 communities. https://BioRender.com/cu1db2k.

To determine how the VOC profiles of plant communities along a diversity gradient are influenced by biotic and abiotic factors, we included data on the diversity of pathogenic oomycetes and arbuscular mycorrhizal fungi (AMF), leaf feeding by herbivores, leaf area index, and soil temperature in the communities studied, and created a structural equation model (SEM; *SI Appendix*, Fig. S7). The model shows a good fit (Fischer’s C = 26.78, *P* = 0.80, AIC =378.43, Fig. 4) and explained a high proportion of the total variance in VOC emission and VOC richness (R^2^_m/c_ = 0.41/0.83 and R^2^_m/c_ = 0.56/0.56, respectively). Including “collection date” as a random factor improved the explained variance for VOC emission but not for VOC richness. With increasing plant species richness, VOC emissions increased both directly and indirectly through a higher proportion of grass biomass. In contrast, while species richness had a positive effect on VOC richness, this effect was partly weakened by indirect negative effects through increased LAI, and reduced soil pathogen diversity (oomycota diversity). Phylogenetic plant diversity contributed again little to the VOC profiles but did promote soil pathogen diversity, thereby increasing VOC richness. Additionally, the LAI played a significant role in directly and indirectly shaping VOC richness in the community, through its influence on herbivore damage. Although herbivore damage increased with plant species richness, it tended to be negatively correlated with VOC emission and richness. Soil temperature and arbuscular mycorrhizal fungi diversity did not significantly influence VOC profiles at the community level (Fig. 4 and *SI Appendix*, Table S5).

### *P. lanceolata* VOC Profiles Are Shaped by the VOCs of the Surrounding Community.

We next turned our attention to the pattern of VOC diversity at the level of a single species. From the 20 communities surveyed on the plant diversity gradient, we selected the ones where *P. lanceolata* is a resident species (eight communities) and collected VOCs in four *P. lanceolata* individuals per community. The actual plant species richness and phylogenetic diversity in the community were positively correlated with the originally sown plant species richness (richness: R^2^ = 0.68, *P =* 0.013; phylogenetic diversity: R^2^ = 0.90, *P <* 0.001).

**Fig. 5.**
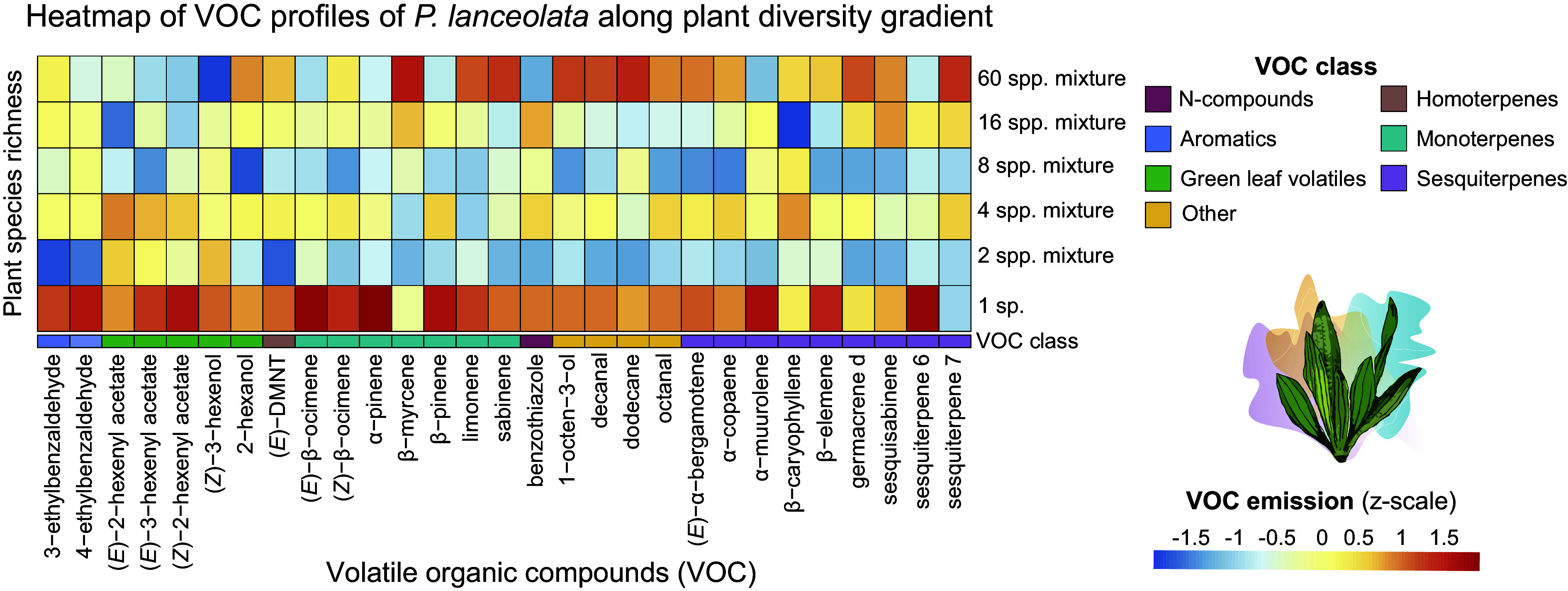
VOC profiles of *Plantago lanceolata* across the plant diversity gradient. Heatmap showing the relative mean abundances (z-scaled per compound across treatment) along the plant species richness gradient spanning from *P. lanceolata* monoculture to 60-species mixtures. Each column corresponds to a specific VOC compound.

**Fig. 6.**
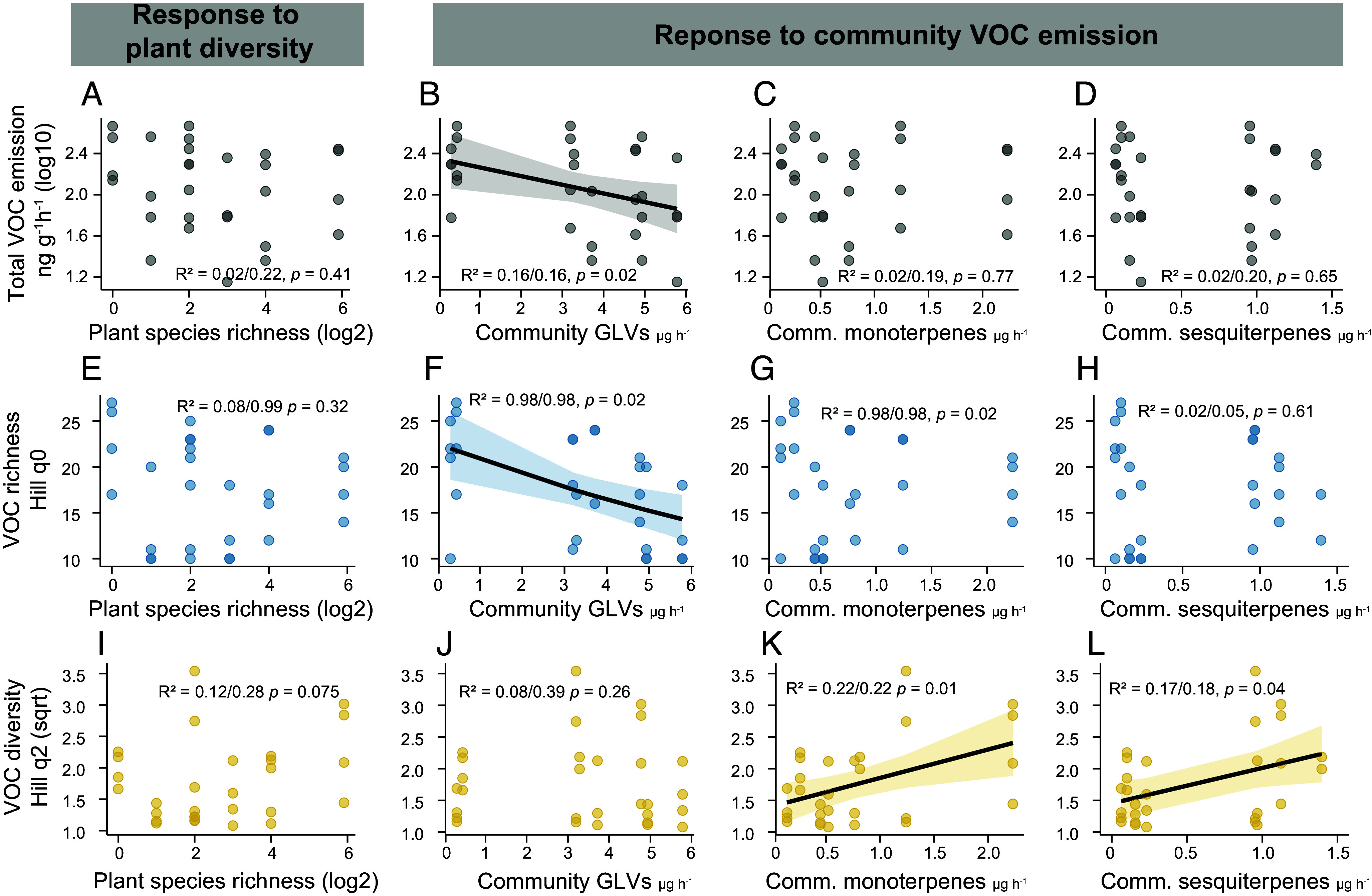
VOC emission and diversity of *P. lanceolata* in response to plant diversity and community VOC emission. Headspace VOC emission of resident individuals of *P. lanceolata.* (*A*–*D*) Total emission (gray, ng per g of fresh weight per hour) across (*A*) sown species richness, and the emission of (*B*) green leaf volatiles (GLVs), (*C*) monoterpenes and (*D*) sesquiterpenes in the surrounding community. (*E*–*H*) VOC richness (blue, Hill q0, number of compounds) across (*E*) sown species richness, and the emission of (*F*) GLVs, (*G*) monoterpenes and (*H*) sesquiterpenes in the surrounding community. (*I*–*L*) VOC diversity (yellow, Hill q2, Simpson diversity) across (*I*) sown species richness, and the emission of (*J*) GLVs, (*K*) monoterpenes and (*L*) sesquiterpenes in the surrounding community. The lines show significant relationships (*P* < 0.05). Sample size = 29 individuals.

We identified 29 VOCs emitted from *P. lanceolata*, classified into GLVs (5) and other fatty acids derivatives (4), aromatics (2), nitrogen-containing compounds (1), homoterpenes (1), monoterpenes (7), and sesquiterpenes (9) (Fig. 5 and *SI Appendix*, Table S2). Overall, the total emission and diversity of VOCs from *P. lanceolata* were not significantly influenced by the plant species richness of the surrounding community (Fig. 6 *A*, *E*, and *L* and *SI Appendix*, Table S6). However, we found that the emission of the GLVs, (*Z*)-3-hexanol and (*Z*)-2-hexenyl acetate, and the monoterpenes, α-pinene, and (*E*)*-*β-ocimene, decreased with the increase of plant species richness in the community (*SI Appendix*, Table S7).

When we investigated how the VOC profiles of the surrounding community influenced *P. lanceolata* VOC profiles, we observed notable effects. In general, an increase in GLV emissions in the surrounding community led to a decrease in both the total emission and number of VOCs emitted by *P. lanceolata* (Fig. 6 *B* and *F* and *SI Appendix*, Table S8). Specifically, *P. lanceolata* reduced the emission of GLVs, such as (*E*)-3-hexenyl acetate and (*Z*)-2-hexenyl acetate, and decreased the emission of terpenes such as, β-pinene, (*E*)-β-ocimene and α-muurolene; with a trend toward lower α-pinene, (*Z*)-β-ocimene and 4,8-dimethylnona-1,3,7-triene (DMNT) emission (*SI Appendix*, Table S9). With increasing monoterpene and sesquiterpene emissions in the surrounding community, the VOC Simpson (Hill q2) diversity in *P. lanceolata* increased (Fig. 6 *K* and *L*), which means that its VOC profiles became more even across compounds. This shift was primarily driven by the increased emission of (*E*)-2-hexenyl acetate, β-myrcene, α-muurolene, and germacrene D, and the decreased emission of (*Z*)-3-hexenol and (*Z*)-2-hexenyl acetate (*SI Appendix*, Table S9). Vegetation height of the surrounding community did not significantly influence *P. lanceolata* VOC emission or its diversity, but total GLV emission of *P. lanceolata* decreased with increasing vegetation height (*x*^2^ = 4.43, *P* = 0.035), specifically the emission of (*E* and *Z*)-2-hexenyl acetate.

Overall, total leaf damage in *P. lanceolata* decreased with increasing plant species richness (*x^2^* = 5.37, *P* = 0.021; *SI Appendix*, Table S6), driven primarily by a reduction in pathogen damage (*x^2^* = 3.38, *P* = 0.06) rather than by a reduction in herbivore damage (*x^2^* = 0.96, *P* = 0.33). When we compared the VOC profiles of *P. lanceolata* with the type of leaf damage, we found that VOC diversity was not influenced by herbivore leaf damage but rather by pathogen damage. VOC Simpson (Hill q2) diversity decreased with increasing pathogen damage (*x^2^* = 6.47, *P* = 0.011), especially due to a decrease in sesquiterpene emission (*SI Appendix*, Table S10). Notably, herbivore damage decreased when individuals were surrounded by communities emitting a high concentration of GLVs (*x^2^* = 4.19, *P* = 0.04; *SI Appendix*, Table S8), while pathogen damage decreased when individuals were surrounded by communities emitting high terpene concentrations (monoterpenes*: x^2^* = 7.36, *P* = 0.007; sesquiterpenes: *x^2^* = 14.25, *P* < 0.001; *SI Appendix*, Table S8).

## Discussion

In this study, we investigated how plant VOC emission and diversity varied across a plant diversity gradient, examining emission from both a whole plant community and from single individuals of *P. lanceolata.* Our results show that VOC diversity increased with increasing plant diversity at the community level, and the VOC emission of an individual plant species was affected more by the emission of the surrounding community than by plant species richness. Furthermore, at the species level, leaf pathogen damage decreased the VOC diversity of *P. lanceolata*. At the community level, plant species richness increased VOC emission via the proportional increase in grass biomass, while its effect on VOC richness was mediated both directly and indirectly through changes in LAI, herbivore damage, and soil pathogens. The results show that the scale of observation is of crucial importance in determining which factors influence plant VOC diversity when a plant diversity gradient is altered.

We show that high-diversity plant communities emit larger amounts and a greater number of VOCs compared to low-diversity communities. This finding supports the hypothesis that the addition of each new plant species to a community enriches the VOC profile by introducing species-specific compounds (26, 38). Notably, most of these compounds were emitted at relatively low concentrations compared to the major compounds present across all communities. Although low in abundance, such minor VOCs can have important ecological functions. Previous studies have shown that even trace-level compounds can play key roles in plant defense, signaling, and mediating interactions with other organisms (39). Thus, the increased presence of these specific, low-abundance VOCs in high-diversity communities may translate into more complex chemical communication both within and between species. Contrary to our expectations, neither phylogenetic diversity nor differences in species composition significantly influenced community VOC profiles, which is consistent with the fact that certain compounds are emitted broadly across taxa (40). Whether and how certain plant species can significantly alter the VOC profiles of the community needs to be further investigated.

Although we expected VOC richness to rise with increasing species diversity, the observed increase in emissions likely reflects not only a greater number of compounds but also higher plant biomass, as diverse communities typically enhance aboveground productivity (41). Yet, total biomass alone did not explain VOC emissions; instead, emissions were strongly enhanced by the proportion of grass biomass in the community, without significantly affecting the VOC richness. Although plant species differ in their VOC emission profiles, our data do not allow us to attribute the higher emissions solely to grasses. The positive relationship between VOC emissions and grass proportion could reflect direct effects if grasses inherently emitted more VOCs than legumes or herbs. However, evidence for this is limited and largely confined to studies at the level of individual plants, some of which even report the opposite pattern (42). Alternatively, grasses may increase emissions at community level indirectly by altering the VOC production of neighboring species. For instance, it has been shown that the legume *Trifolium pratense* increases its VOC emission when growing alongside *Dactylis glomerata* grass. This is possibly a response to interspecific competition with the grass (38). Further studies are needed to clarify the mechanisms by which grasses may shape VOC emissions at plant community level, whether through their own emission, competitive interactions with neighboring species, or other indirect pathways such as shifts in herbivore or pathogen pressure.

Our β-diversity analysis revealed that VOC profile dissimilarity increased with plant diversity in a community. As plant species richness increased, community-level VOC differences shifted from being primarily driven by compound nestedness (the presence or absence of compounds) to being driven by compound replacement (changes in the identity of emitted compounds). This suggests that there is a point where the total number of compounds stabilizes, but the identity of those compounds varies significantly. Interestingly, 37% of the VOCs were ubiquitous across all plant diversity levels studied, including compounds like (*Z*)-3-hexen-1-ol, α-pinene, (*E*)-β-caryophyllene, and methyl salicylate, which are often constitutively emitted or have their emission frequently induced by environmental stimuli from many plant species (43). These compounds play important roles in indirect defense against herbivores and pathogens, as well as in defense priming, both intra-and interspecifically, by inducing defense compounds in neighboring plants (44, 45). This variation in VOC composition, driven by plant diversity, may influence biotic interactions and structure in the community (46, 47), emphasizing the potential role of VOC diversity in biodiversity-ecosystem functioning relationships. Assessing the ecological impacts of compound enrichment or replacement in VOC bouquets, however, can be a challenge, especially at the community level, as VOC functions may vary between species and organ type (48). Furthermore, plants emit and perceive info-chemicals simultaneously, yet how these interactions change across different ecological scales remains largely unexplored, especially under field conditions.

As mentioned before, both antagonistic and mutualistic organisms can shape plant VOC emissions (9, 49, 50). More importantly, in diverse plant communities, there is often an accumulation of mutualists and a dilution of antagonists, both above- and belowground (20, 28, 51). These shifts can drive individual plants to emit distinct VOC profiles in low- *versus* high-diversity communities due to varying pressures (5). However, most existing studies have focused on species-level interactions, and scaling up plant VOC-mediated interactions to the community level can be a challenge. Increasing chemical diversity in the community can be positively correlated with the abundance of arthropods and reduce leaf damage (52). In this study, an increase in plant species richness shaped VOC profiles in the community through both direct and indirect routes. While VOC emission was directly enhanced by higher plant species richness and grass biomass proportion rather than biotic factors, VOC richness was influenced not only directly by plant species richness but also indirectly by LAI and soil microbiota. VOCs are thus influenced by community characteristics in different ways. Plant diversity directly increased VOC emissions, but its influence on VOC diversity is also evident in changes in abiotic and biotic factors that can either promote or limit the number of compounds emitted. Notably, although only a trend, we detected a negative association between herbivore damage and VOC profiles, suggesting that communities emitting higher amounts and a greater number of VOCs tended to experience less herbivore damage, potentially indicating a repellent effect. However, this interpretation should be treated with caution, as our VOC measurements are only a snapshot, while measurements of damage caused by herbivores may represent cumulative damage to plants over a longer period of time. Furthermore, as many VOCs produced by plants can also be emitted by other organisms, such as microbes or insects (53, 54), we should be aware that the VOC bouquet we sampled may not be exclusively plant-derived. Temporal dynamics can influence community-level VOC profiles even over short periods, primarily affecting compound abundance rather than identity. Consistent with this, including collection date as a random factor improved model performance for total emission, likely reflecting environmental drivers like light, temperature, and atmospheric conditions.

Plants are known to respond to VOCs emitted by their neighbors (55), and this response can be influenced by the identity of the neighboring plants and the diversity of the surrounding community (5, 38, 56). In this study, plant diversity did not directly affect the VOC emission of resident *P. lanceolata* individuals, but plant diversity influenced the VOC emission of plant communities, which in turn influenced the VOC emission of *P. lanceolata* within them. Our findings add to previous studies on plant–plant communication (21, 22, 38, 56) by demonstrating that VOC emissions of receiver plants are influenced not only by individual neighbors but also by the overall VOC profile of the community. The observed decrease in total VOC emissions of *P. lanceolata* in communities with high concentrations of GLVs, together with their increased VOC diversity in communities dominated by terpenoids, may be attributed to several underlying mechanisms. For instance, 1) plants might prioritize alternative defensive strategies in “noisy” VOC environments, where signaling is less effective; 2) they might allocate fewer resources to VOC emission when benefitting from the protective effects of VOCs released by other plants in the community; 3) the emission of fewer VOCs may reduce the risk of eavesdropping by neighboring plants, thereby enhancing the defensive advantage of the emitter; or 4) these changes could represent adaptive responses to specific VOC cues conveying different kinds of information. Since the diversity of terpene volatiles emitted by plants is often far greater than the diversity of GLVs, terpenoids may transmit more distinct information (48, 57, 58). Certainly, further research is needed to fully understand the basic functions of plant–plant communication and how this can vary across diversity gradients.

In this study, *P. lanceolata* individuals experienced lower herbivore damage in communities emitting high concentrations of GLVs, and pathogen damage decreased when individuals were surrounded by communities emitting high terpene concentrations. These results suggest that plants exhibit specific responses to distinct VOCs in the community (59). The surrounding VOC composition can influence *P. lanceolata* performance by direct effects or by modifying plant defense strategies. For instance, exposure to GLVs and terpenes from neighboring plants not only alters the VOC emission patterns of receiver plants (60) but also induces the accumulation and biosynthesis of defensive compounds, reducing herbivore and pathogen damage (61–63). Moreover, at the community level, VOCs function as long-distance signals, attracting or repelling organisms attempting to enter the community, while either facilitating or disrupting communication among plant species (56). Increased VOC emissions and diversity may disrupt cues used by specialist herbivores or pathogens, benefitting plants through “chemical noise”(64), but greater VOC complexity might also hinder interactions with mutualists like pollinators or parasitoid wasps (10, 65). Surrounding VOCs could also have direct effects on pathogens or herbivores in sufficient concentrations since many of these compounds are known to be toxins or feeding deterrents (66). Overall, VOC profiles might play a crucial role in shaping biotic interactions, directly enhancing plant defenses and indirectly influencing ecological dynamics.

Our findings show that plant diversity shapes both the structural and chemical dimensions of ecosystems, influencing plant interactions with their environment and other organisms. We reveal that higher species richness indirectly increases VOC emissions through greater grass biomass and enhances VOC diversity via shifts in biotic interactions. Community-level VOCs also affect individual plant emissions and leaf damage, underscoring the role of VOC diversity in plant communication, trophic interactions, and ecosystem functioning. Further research should clarify how community-level VOCs influence plant performance through direct or plant-mediated mechanisms.

## Materials and Methods

### Field Site and Experimental Design.

This study was conducted in “The Jena-Experiment” (50°55′ N, 11°35′ E; 130 m a. s. l.), a long-term grassland biodiversity experiment in Jena, Germany (35). Since 2002, the plant species community compositions, selected from a pool of 60 native grassland species, have been maintained through regular weeding (2 to 3 times per year) and mowing (twice yearly) to replicate traditional management of Central European grasslands. We selected 20 communities (plots) representing a gradient in species richness, ranging from monocultures to 60 plant species-mixtures (1, 2, 4, 8, 16, and 60 plant species), as well as different functional group richness (1 to 4 functional groups: grasses, small herbs, tall herbs, and legumes). Among the 20 communities studied, *P. lanceolata* L. (ribwort plantain) belonged to the sown species combinations in eight communities. At the community level, we measured VOC emission, realized plant species richness, aboveground biomass, vegetation height, leaf area index, soil temperature, herbivore damage, and soil microbiota (oomycota and arbuscular mycorrhiza fungi). At the species level (*P. lanceolata*), we measured VOC emission, aboveground biomass, and percentage of leaf damage by herbivores and pathogens. All the measurements were done between 25 May and 10 June 2021, approximately 1.5 mo after the most recent weeding campaign.

### Headspace VOC Collection.

VOC emission at the community and species level was measured using a push–pull system for 2 h (*SI Appendix*, Fig. S8). At the community level, three cylindrical frames (Ø 50 cm x 80 cm) covered with PET films (sealed with a film sealing machine FERMANT® 120 N) were installed randomly in the core area (3 x 3 m) of each community. At the species level, four individuals of *P. lanceolata* were individually enclosed with PET bags (Toppits® Bratschlauch, Melitta, Minden, Germany) tightened on top and at the bottom with a cable binder. A small sponge was used between the bag and the cable binder, to reduce potential damage of the plant. In both systems, charcoal-filtered air was continuously pumped into these cages or bags (Community level: 2 L/min flow rate, species level: 1 L/min). At the same time, air was pumped out through VOC traps, consisting of 25 mg of Porapak absorbent (ARS, Grainville, FL) inserted in Teflon tubes (Community level: 1.0 L/min flow rate, species level: 0.7 L/min). All volatile collections were performed between 9:00 am and 1:00 pm. After VOC collection, the VOC traps were eluted with 200 µL dichloromethane containing nonyl acetate as an internal standard (SigmaAldrich, 10 ng µL^−1^). VOCs were analyzed using a gas chromatograph coupled to a mass spectrometer (GC-MS) with helium as the carrier gas for compound identification (Hewlett-Packard 6890 gas chromatograph coupled with a Hewlett-Packard 5973 mass spectrometer) and a gas chromatograph coupled to a flame ionization detector (GC-FID) with hydrogen as the carrier gas for compound quantification [details in (67)]. VOCs were identified by comparing retention times and mass spectra to those of authentic standards obtained from Fluka (Seelze, Germany), Roth (Karlsruhe, Germany), Sigma (St. Louis, MO) or Bedoukian (Danbury, CT), or to reference spectra in the Wiley and National Institute of Standards and Technology libraries (NIST). The quantity of each compound was determined from its peak area in the FID trace in relation to the area of the internal standard using the effective carbon number concept (68).

### Community Plant Trait Measures.

In each community, we measured species-specific plant cover (% per species), plant aboveground biomass inside the VOC cages (fresh weight), plant community mean height and leaf area index (LAI). Percentage cover per species was estimated in a 3 m × 3 m area of the community using a decimal scale (69), including all target species (sown in a particular community) and all plant species, which colonized the communities from the surroundings. Community leaf area index (LAI) was measured using a portable LAI-2200C plant canopy analyzer (LI-COR, Lincoln) at ten randomly selected positions within each plot. Community mean height was calculated by averaging the vegetation height (highest leaves) measured at ten randomly selected spots in each plot. Based on the realized plant species richness and vegetation cover, we calculate plant taxonomic, and phylogenetic diversity.

### Community Herbivore Damage.

Herbivory damage by invertebrates and small mammals was quantified as the proportion of damaged leaf area relative to the total leaf area for each plant species within the community. To estimate community-level herbivory, a weighted mean of species-specific herbivory values was calculated for each plot, using leaf biomass as the weighting factor (details in 51, 70).

### Community Soil Microbiota Diversity.

Soil Oomycota and arbuscular mycorrhizal fungi diversity were estimated at the community level from bulk soil collected in each studied community during the same period of VOC collection. Oomycota and AMF diversity were obtained from Solbach and Bonkowski (71) and Albracht et al. (28, 72), and then transformed to Hill numbers (Hill q1). Briefly, DNA was extracted from bulk soil (pooled and homogenized from 4 random soil cores (4 cm diameter, 5 cm depth) per community) with the Quick-DNA Fecal/Soil Microbe Miniprep Kit (Zymo Research Europe GmbH, Freiburg, Germany). The ITS1 region of Oomycota was then amplified with barcoded primers applying the procedure described in Fiore-Donno and Bonkowski (73). Sequencing was performed with a MiSeq v3 Reagent kit of 600 cycles on a MiSeq Desktop Sequencer (Illumina Inc., San Diego, CA) at the Cologne Center for Genomics (CCG, Cologne, Germany). The raw sequence reads were assembled and quality-filtered in *mothur* (74). Oomycota diversity (Shannon diversity) was estimated with *vegan* (75). For AMF, the SSU rRNA region was amplified with nested PCR (primer pairs WT0/Glomer1536 and NS31/AML2) and sequenced at the Illumina MiSeq platform at the Soil Ecology department of Helmholtz Centre for Environmental Research (Halle (Saale), Germany). Sequencing data were processed with the dadasnake pipeline (76). AMF diversity (Shannon diversity) was estimated with *phyloseq* (77) on rarefied data (2400 reads per sample).

### Measurement of Soil Temperature.

In each plot, the soil temperature was measured at 5 cm depth, using thermometers of a controller area network-bus module system (JUMO). The temperature sensors were lance probes with a diameter of 4.5 mm and a length of 200 mm. The measuring element was a PT100 resistor with a tolerance of ±0.1 °C at 0 °C. The sensor operated in a four-wire connection to the data-acquisition module of the controller area network-bus network (78, 79).

### Data Analysis.

Realized plant diversity was assessed at both the taxonomic and phylogenetic levels using Hill numbers (Hill q0 and q1). Plant species-level cover was used as an abundance variable for diversity calculations (α- and β-diversity). Phylogenetic diversity was calculated by constructing a phylogenetic tree that included all plant species recorded across communities. To examine whether sown species richness was still significantly correlated to the realized plant diversity in the selected communities, we performed a linear regression model with sown species richness (log2 transformed) as the explanatory variable and the different plant diversity indices measured as dependent variables.

To explore the role of plant diversity in shaping VOC emissions, we calculated VOC diversity at both the community and species levels. The α-diversity metrics were used to determine the relationship between the number of VOCs in a bouquet and their relative abundance. We used diversity orders (Hill numbers from 0 to 2) to adjust the sensitivity of diversity metrics to low-concentration compounds (80). The β-diversity metric was used to determine how VOC composition changes across the plant diversity gradient and whether this is due to compound turnover among communities or nestedness. In both cases, VOC emission (ng h^−1^) was used as abundance information. Since the plant communities have different degrees of homogeneity, we summarize the emission of the three replicates of each community and calculate their VOC diversity. At the species level, VOC diversity was standardized by leaf biomass to account for variation in plant size (ng g_fw_ h^−1^).

To evaluate the effects of plant diversity on VOC emission and diversity at community level, we performed mixed-effect regression models, partial Spearman correlation, distance-based redundancy analysis (dbRDA), and SEMs. To test the effect of species diversity or plant biomass at the community level, we included fresh weight biomass as a covariate fitted before the fixed factor (sown plant richness, realized diversity, or proportion of biomass per functional group). Block and collection date were included as random effects (e.g., y ~ biomass + log2 (sown_plant_richness) + (1|block) + (1|collection_date)). To test the effect of plant biomass independently, we corrected VOC emission (ng g^−1^ h^−1^) and VOC richness (number of compounds g^−1^) by biomass and modeled them using sown species richness as the fixed factor, again with block and collection date as random effects (e.g., y ~ log2 (sown_plant_richness) + (1|block) + (1|collection_date)). To assess term significance, we used ANOVA type I sum-of-squares and adjusted p-values for false discovery rate (FDR) due to multiple testing (81). We extracted marginal and conditional R^2^ values from the models to evaluate the proportion of variance explained. When needed, data were transformed to meet the assumptions of normality.

To investigate the relationship between plant species composition and VOC emissions, we performed partial Spearman correlations. Correlations were computed between each VOC and individual plant species, controlling for community diversity (log2-transformed sown richness) as a covariate. P-values were adjusted for multiple testing using the false discovery rate (FDR). VOCs with at least one significant correlation (FDR < 0.05) were retained and visualized in a heatmap (*SI Appendix*, Fig. S3). We further analyzed variation in VOC profiles using distance-based redundancy analysis based on Bray–Curtis dissimilarities of log-transformed emission data (ng h^-1^). The model included sown plant species richness (log2-transformed), the proportion of grass biomass, and a phylogenetically informed species composition as predictors, with collection date as a conditional variable. To estimate phylogenetically informed species composition, we used vegetation cover, and a phylogenetic PCA was performed using a community phylogeny, from which the first axis was extracted. Significance of terms was assessed with permutation tests (999 permutations).

To evaluate whether abiotic and biotic interactions driven by species diversity altered the effects and direction of the relationships on VOC emission and richness, we constructed a SEM fitting mixed-effect models, following the conceptual framework described in *SI Appendix*, Fig. S7. We selected the final model by excluding the nonsignificant factors with limited theoretical support.

At the species level, we fitted either sown species richness, community VOC profiles, or leaf damage as fixed factors and plot nested in block and collection date as random effects [e.g., y ~ log2 (sown plant richness) + (1|block/plot) + (1|collection_date)]. To assess term significance, we used ANOVA type I sum-of-squares and adjusted p-values for false discovery rate (FDR) due to multiple testing (81). We extracted marginal and conditional R^2^ values from the models to evaluate the proportion of variance explained. When needed, data were transformed to meet the assumptions of normality.

All statistical analyses and data visualization were conducted in R version 4.5.1 (82) using the *rBExIS, dplyr*, *tidyverse*, *tibble,* and *janitor* for data retrieval, cleaning, and formatting (83–86). Plant diversity calculations were performed using the *hillR* package (87), and phylogenetic diversity was estimated using the *V.PhyloMaker* package (88), which constructs phylogenies based on the GBOTB backbone (89). VOC diversity metrics were computed using the *vegan*, *hillR*, and *BAT* packages (75, 87, 90). Mixed-effects regression models were implemented using the *lme4*, *lmerTest*, and *glmmTMB* packages (91–93), with model variance components extracted using *MuMIn* (94). Structural equation modeling was conducted using the *piecewiseSEM* package (95). For data visualization, we used *ggplot2*, *ggeffects*, *ComplexHeatmap*, and *pheatmap* (96–99). Illustrations were created in R and BioRender.com, with final graphics refined in Adobe Illustrator CC 2021.

## Supplementary Material

Appendix 01 (PDF)

## Data Availability

The Quarto (R) notebook containing the code used (https://doi.org/10.25829/AR6J-3P78) (100), community VOC profiles (https://doi.org/10.25829/NV3C-BJ47) (101), abiotic and biotic traits measured at the community level (https://doi.org/10.25829/ZP1G-F469, https://doi.org/10.25829/3EMH-HX60) (102, 103), and *P. lanceolata* VOC profiles and leaf damage (https://doi.org/10.25829/GDS4-GH45, https://doi.org/10.25829/PRQ5-CW61) (104, 105) are available through the Jena Experiment Information System (https://jexis.idiv.de).
