# Supplementary material for: Plant diversity influences plant volatile emission with varying effects at the species and community levels
Source: Proc Natl Acad Sci U S A. 2026 Jan 15;123(3):e2518326123. doi: 10.1073/pnas.2518326123 (PMC12818445; doi:10.1073/pnas.2518326123)
Supplement: Supplementary file 1 — Appendix 01 (PDF) [file pnas.2518326123.sapp.pdf]

PNAS

Supporting Information for

Plant diversity influences plant volatile emission with varying effects at the species and community levels

Pamela Medina-van Berkum[1,12*], Cynthia Albracht[2,3,4], Maximilian Bröcher[5], Marcel Dominik Solbach[6], Gideon Stein[7,8], Michael Bonkowski[6], François Buscot[3,7], Anna Heintz-Buschart[2], Anne Ebeling[5], Nico Eisenhauer[8,9], Tarek S. El-Madany[10], Yuanyuan Huang[8,9], Karl Kuebler[10], Sebastian T. Meyer[11], Jonathan Gershenzon[1], Sybille B. Unsicker[1,12*]

[1] Department of Biochemistry, Max Planck Institute for Chemical Ecology, Jena, Germany

[2] Biosystems Data Analysis, Swammerdam Institute for Life Sciences, University of Amsterdam, Amsterdam, The Netherlands.

[3] Department Soil Ecology, Helmholtz Centre for Environmental Research—UFZ, Halle (Saale), Germany.

[4] Institute for Biosafety in Plant Biotechnology, Julius Kühn Institute, Quedlinburg, Germany.

[5] Institute of Biodiversity, Ecology and Evolution, University of Jena, Jena, Germany.

[6] Terrestrial Ecology Group, Institute of Zoology, University of Cologne, Cologne, Germany.

[7] Computer Vision Group, Faculty of Mathematics and Computer Science, University of Jena, Jena, Germany.

[8] German Centre for Integrative Biodiversity Research (iDiv), Halle-Jena-Leipzig, Leipzig, Germany.

[9] Institute of Biology, Leipzig University, Leipzig, Germany.

[10] Max Planck Institute for Biogeochemistry, Jena, Germany.

[11] Terrestrial Ecology Research Group, Department of Ecology and Ecosystem Management, School of Life Sciences Weihenstephan, Technical University of Munich, Munich, Germany.

[12] Plant-Environment-Interactions Group, Botanical Institute, Kiel University, Kiel, Germany.

Corresponding authors: Pamela Medina-van Berkum, email: pberkum@ice.mpg.de; Sybille B. Unsicker, email: sunsicker@bot.uni-kiel.de

This PDF file includes:

Figures S1 to S8
Tables S1 to S10
References

- **Figure S1.** Percentage of vegetation cover across the studied communities in the Jena Experiment during the summer of 2021.
- **Figure S2.** Realized plant species diversity of the communities studied across a diversity gradient in the Jena Experiment during the summer of 2021.
- **Figure S3.** Tripartite Sankey diagram showing the flow from plant species biomass within each community (left) to total community biomass (middle) and to the abundance of volatile organic compounds (VOCs) emitted by those communities (right).
- **Figure S4.** Beta diversity of plant species and volatile organic compounds at community level across a plant diversity gradient.
- **Figure S5.** Volatile organic compound (VOC) emission (ng g^{-1} h^{-1}) and diversity at the community level across the plant diversity gradient.
- **Figure S6.** Heatmap of partial Spearman correlations between plant species and volatile organic compounds emission in the community, controlling for sown species diversity as a covariate.
- **Figure S7.** Conceptual framework of the direct and indirect effects of plant species diversity on plant volatile organic compound emission in an experimental grassland field
- **Figure S8.** Graphical illustration of the headspace push-pull systems for volatile organic compounds collection used in the field.
- **Table S1.** Plant species in the communities studied in the Jena Experiment in May 2021.
- **Table S2.** List of volatile compounds identified at plant community level and species level.
- **Table S3.** Community level: Wald-chi-squared analysis of variance (ANOVA) results for the linear mixed-effects models testing effects of plant diversity on VOC emission and diversity at community level.
- **Table S4.** Community level: Wald-chi-squared analysis of variance (ANOVA) results for the linear mixed models testing effects of plant diversity on each VOC emitted at community level.
- **Table S5.** Community level: Path coefficient for the fitted final structural equation model to investigate the direct and indirect effects of plant species richness on plant volatile profiles in an experimental grassland field.
- **Table S6.** Species level: Wald-chi-squared analysis of variance (ANOVA) results for the linear mixed models testing the effects of plant diversity on *Plantago lanceolata* VOC emission and leaf damage.
- **Table S7.** Species level: Wald-chi-squared analysis of variance (ANOVA) results for the linear mixed models testing the effects of plant diversity and leaf damage on each VOC emission of *Plantago lanceolata*.

- **Table S8.** Species level: Wald-chi-squared analysis of variance (ANOVA) results for the linear mixed models testing the effects of Community VOC on *Plantago lanceolata* VOC emission and leaf damage.
- **Table S9.** Species level: Wald-chi-squared analysis of variance (ANOVA) results for the linear mixed models testing the effects of Community VOC on each VOC emission of *Plantago lanceolata.*
- **Table S10.** Species level: Wald-chi-squared analysis of variance (ANOVA) results for the linear mixed models testing the effects of leaf damage on *Plantago lanceolata* VOC emission.

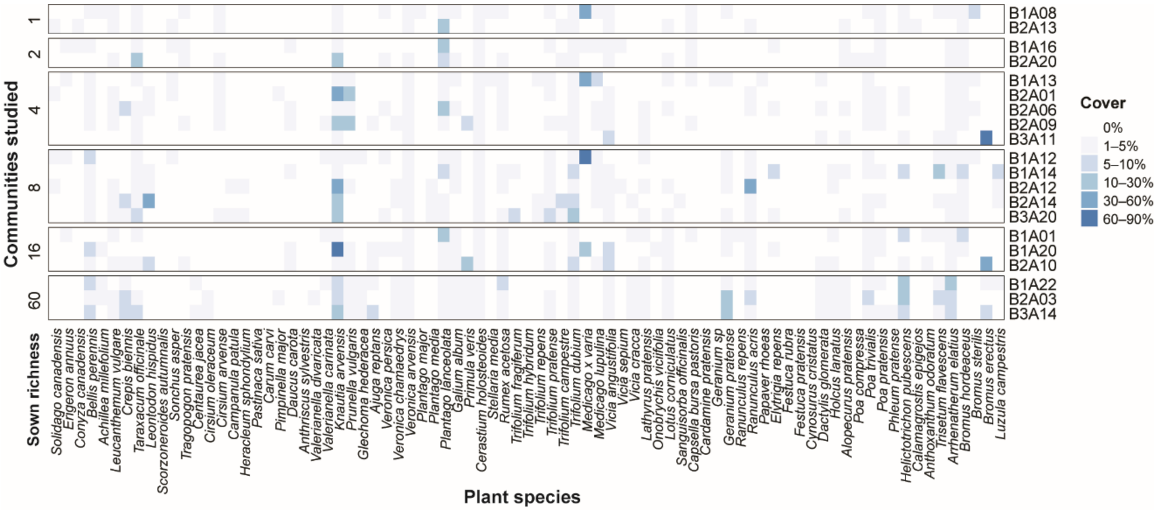

Figure S1. Percentage of vegetation cover across the studied communities in the Jena Experiment during the summer of 2021. Percentage cover per plant species was estimated in a 3m x 3m area of the community using a decimal scale, including all target species (sown in a particular community) and all plant species, which colonized the communities from the surroundings). A total of 82 plant species were identified.

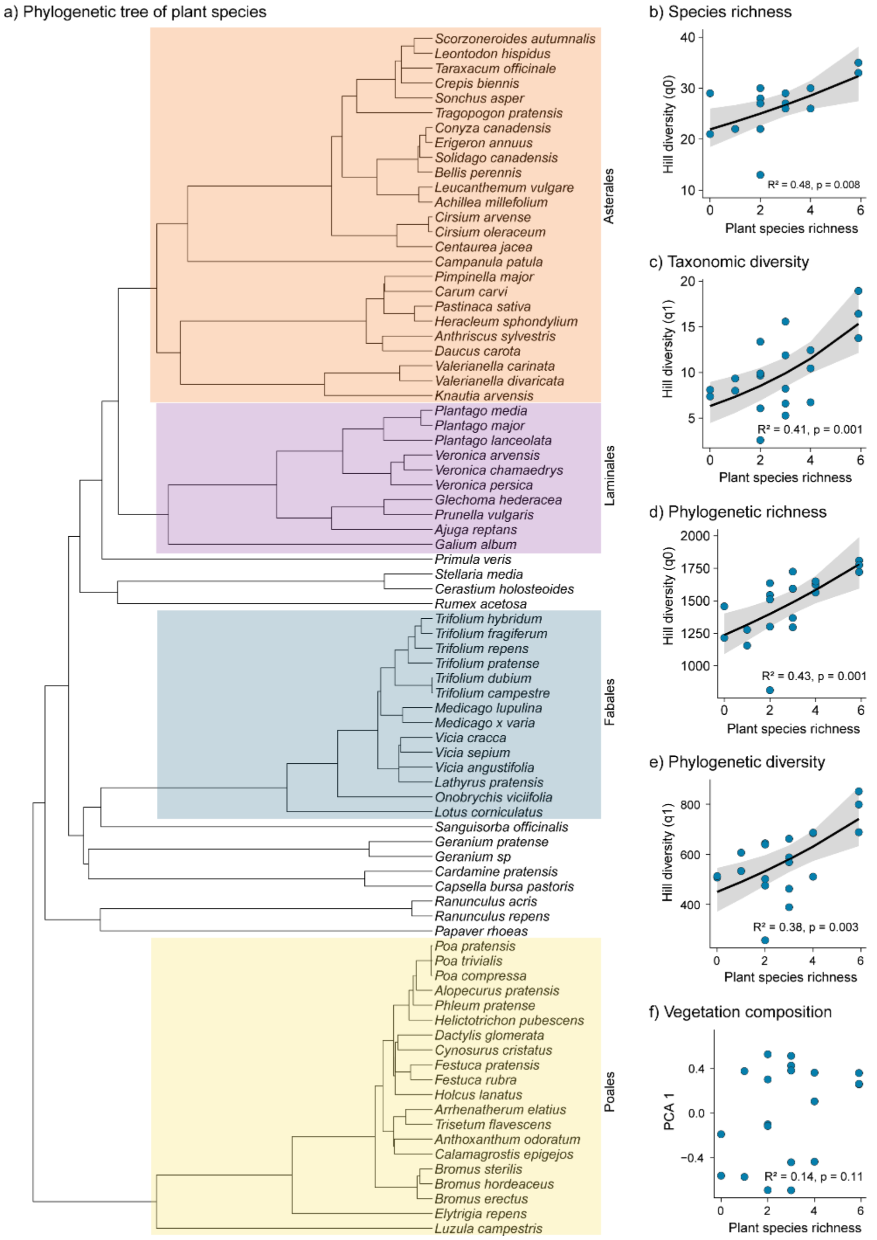

Figure S2. Realized plant species diversity of the communities studied across a diversity gradient in the Jena Experiment during the summer of 2021. a) Phylogenetic tree of plant species identified in the communities studied. A total of 81 species were identified. Major families are highlighted: Poales (yellow), Fabales (blue), Lamiales (purple), and Asterales (orange). Regression models (linear or Poisson) testing the relationship between sown species richness and b) realized species richness (Hill q0), c) Shannon diversity (Hill q1, based on vegetation cover), d) phylogenetic richness (Hill q0, based on phylogenetic relationships), e) phylogenetic diversity (Hill q1, based on vegetation cover and phylogenetic relationships) and vegetation composition based on PCA1. Sample size = 20 communities.

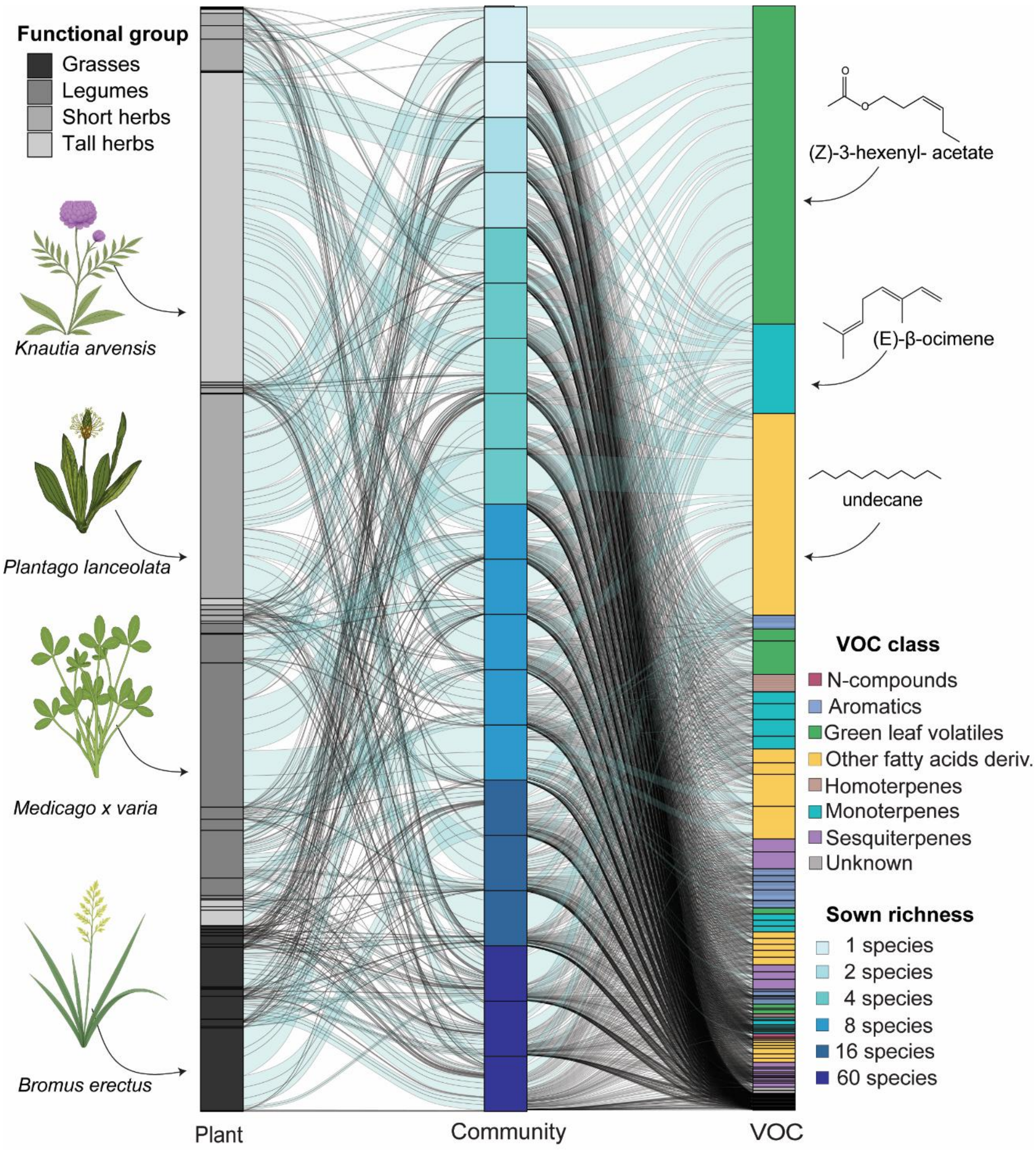

Figure S3. Tripartite Sankey diagram showing the flow from plant species biomass within each community (left) to total community biomass (middle) and to the abundance of volatile organic compounds (VOCs) emitted by those communities (right). The width of each connection is proportional to the relative biomass or compound abundance. Figures in the plot was created with Biorender.com and ChemDraw.

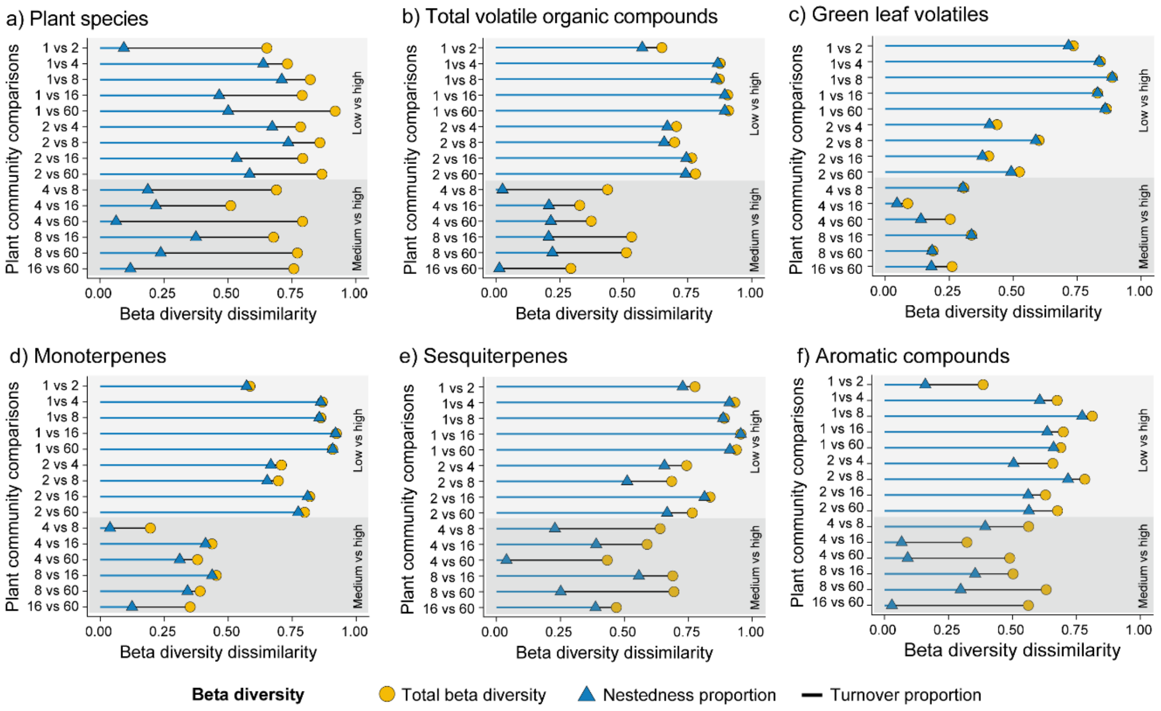

Figure S4. Beta diversity of plant species and volatile organic compounds emitted at community level across a plant diversity gradient. Pairwise comparison of a) plant species, b) total volatile profiles, c) green leaf volatiles, d) monoterpenes, e) sesquiterpenes and f) aromatic compounds between communities of different levels of diversity. Beta diversity was estimated by using the vegetation cover per plant species and the mean of emission (µg h^{-1}) per compound per diversity level.

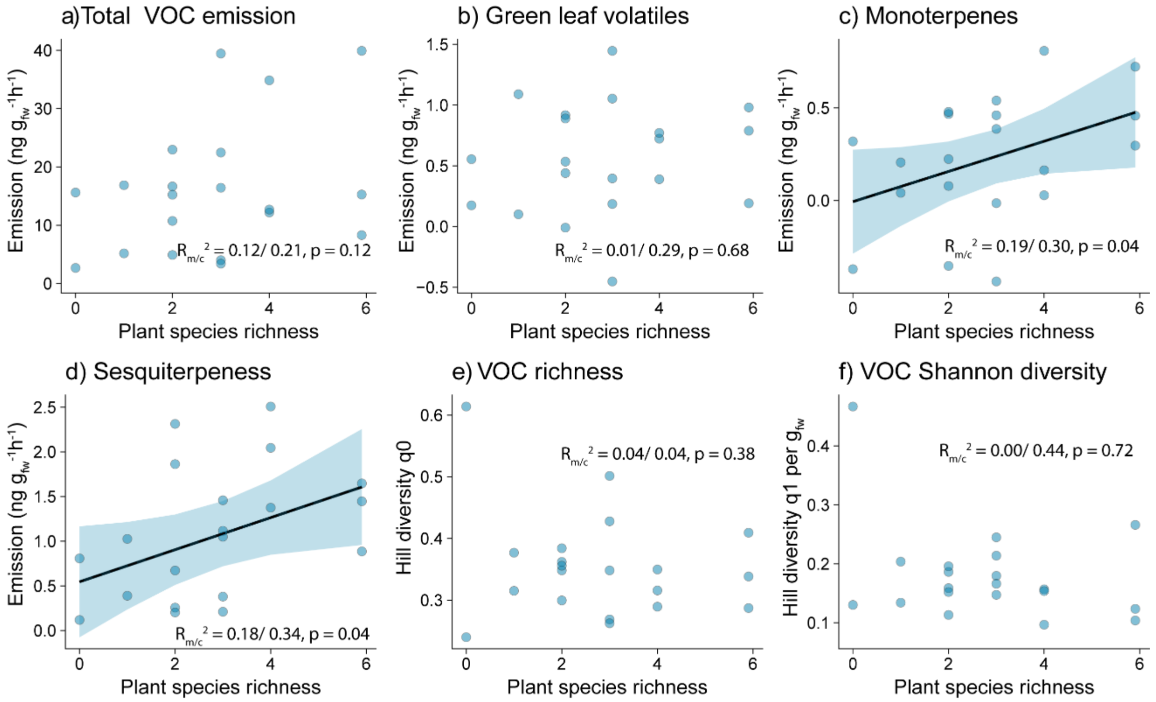

Figure S5. Volatile organic compound (VOC) emission (ng g^{-1} h^{-1}) and diversity at the community level across the plant diversity gradient. Headspace VOC emission of experimental grassland communities across a plant diversity gradient ranging from monocultures to 60-species mixtures. (a-d) VOC emission (ng per gram fresh aboveground biomass per hour) at the community level, including a) total volatiles, b) green leaf volatiles, c) monoterpenes, and d) sesquiterpenes. (e-g) VOC diversity at the community level, including e) richness (Hill q0, number of compounds), and f) Shannon diversity (Hill q1) across the diversity gradient. Sample size = 20 communities, with each data point representing the sum of VOC profiles from three random positions within each community. The lines show significant relationships (p < 0.05).

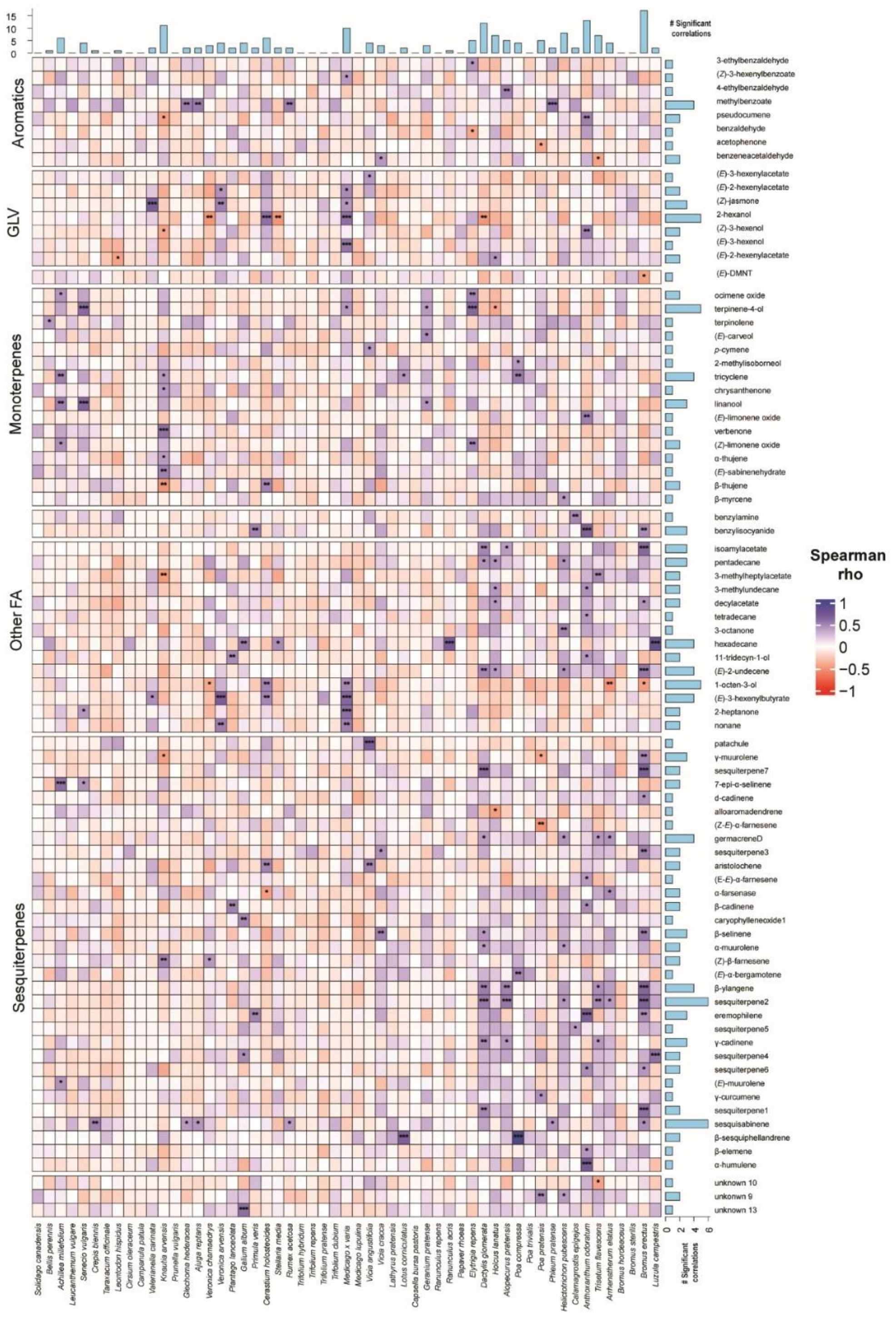

Figure S6. Heatmap of partial Spearman correlations between plant species and volatile organic compounds emission in the community, controlling for sown species diversity as a covariate. Biomass (g FW) and VOC emission (ng h^{-1}) were measured per cage (replicates, 3 per community) and scaled. P-values were FDR-adjusted; only VOCs with at least one significant correlation are shown. Asterisks indicate significance (**p* < 0.05; ***p* < 0.01; ****p* < 0.001). Bar plots show the total number of significant correlations per plant (top) and per VOC (side).

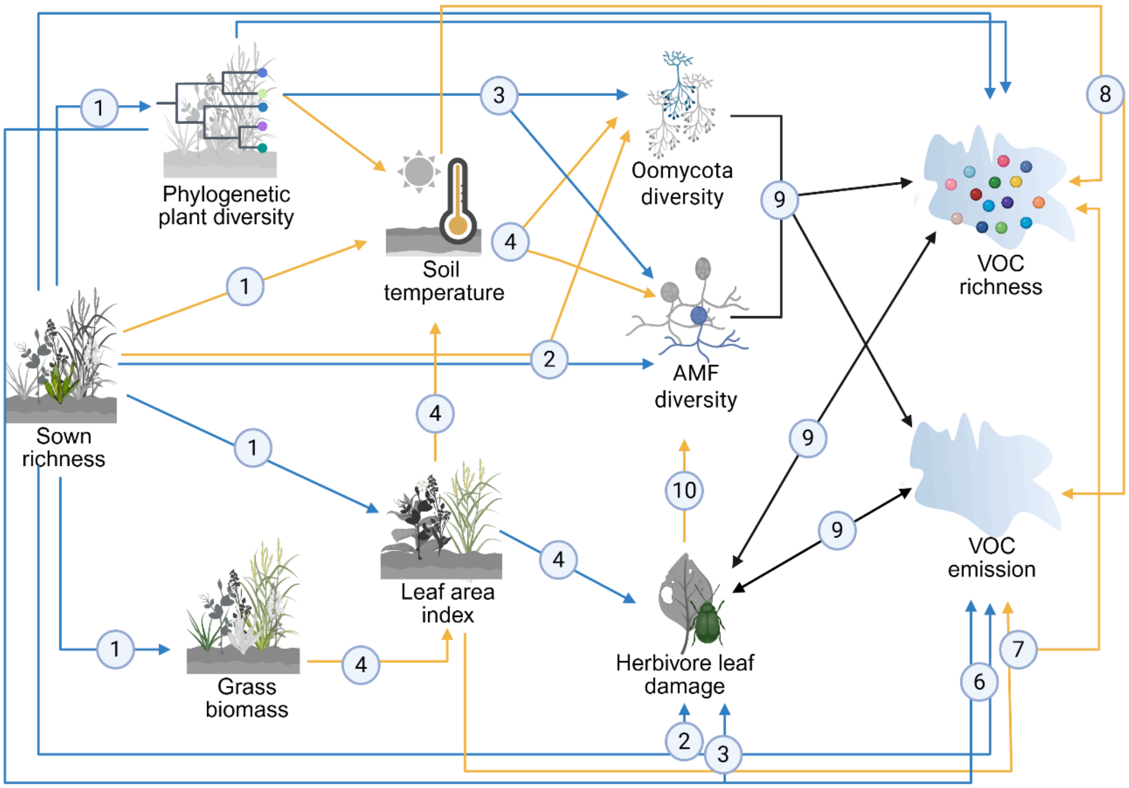

Figure S7. Conceptual framework of the direct and indirect effects of plant species diversity on plant volatile organic compound emission in an experimental grassland field. Blue arrows represent positive effects, while yellow arrows denote negative effects**.** The conceptual model is based on hypotheses derived from literature. 1) Sown plant species richness will promote phylogenetic diversity in the community, grass biomass, leaf area index, and reduce soil temperature (1, 2). 2). Sown plant species richness will promote AMF diversity and herbivore leaf damage but will reduce soil-borne plant pathogen diversity (3, 4). 3) Plant phylogenetic diversity will increase soil pathogens and AMF diversity and reduce herbivore damage (5, 6). 4) Leaf area index will increase herbivore damage and reduce soil temperature and be reduced by grass biomass. 5) Soil temperature will reduce AMF and soil pathogens diversity. 6) The increase of sown species richness and plant phylogeny diversity will promote community VOC richness and emission (7). 7) Leaf area index will reduce VOC richness and emission (8). 8) Soil temperature will increase VOC richness and emission (8). 9) Herbivore damage, soil pathogens, and AMF diversity will affect VOC emission and richness, but also VOC profiles will affect herbivore damage (8). 10) Herbivore reduce AMF diversity (9). https://BioRender.com/xwl4fgw.

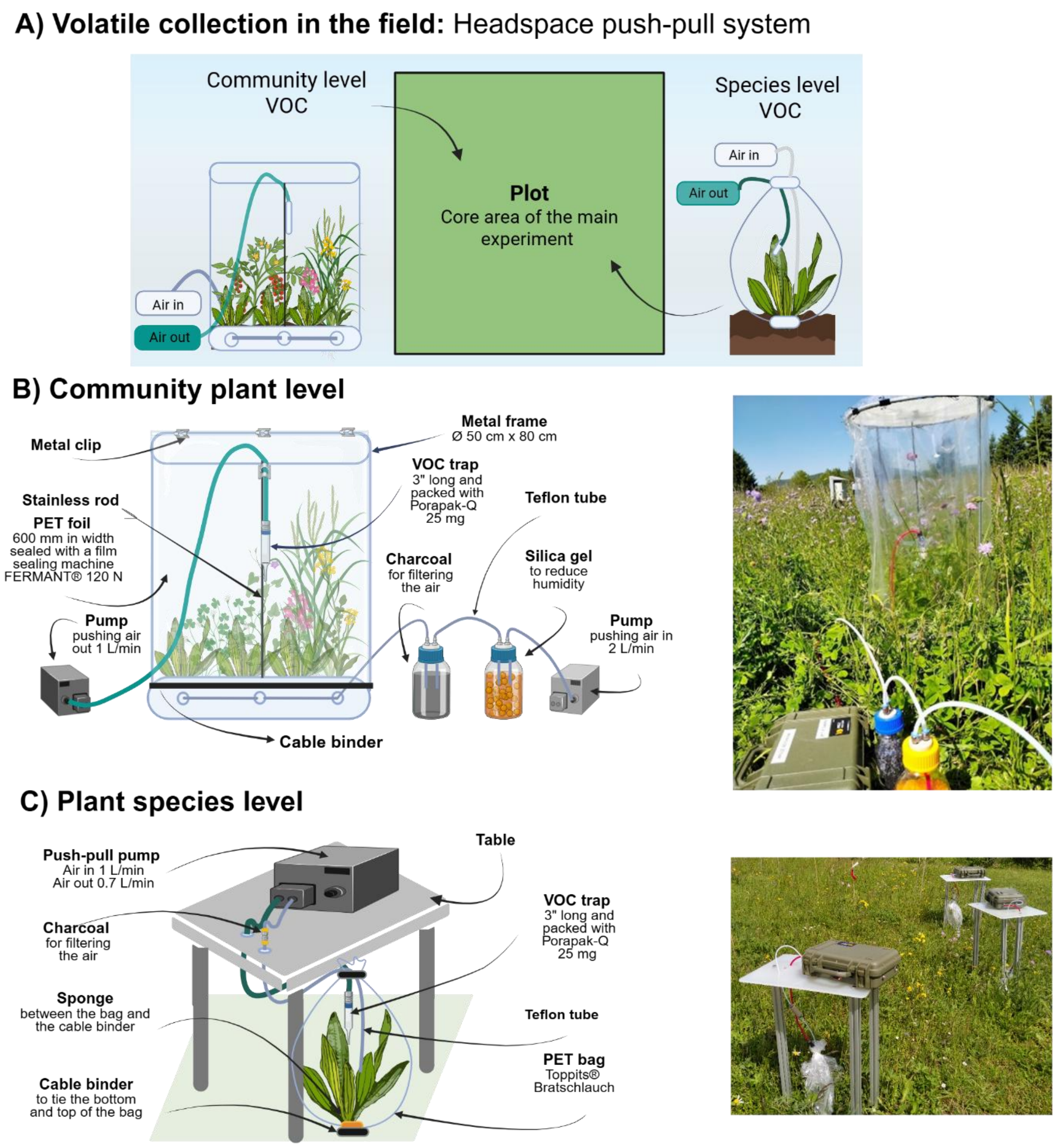

Figure S8. Graphical illustration of the headspace push-pull systems for volatile organic compounds collection used in the field. A) Headspace push-pull system was used for VOC collection at the community and species levels. B) Community-level VOC system, C) Species-level system. VOC collection was performed in the morning for 2 hrs. https://BioRender.com/ohjglbb.

Table S1. Plant species in the communities studied in the Jena Experiment in May 2021. Plant species in the communities studied and whether they were present or not inside the community volatile collection cages. Plant species type represents whether they are part of the species pool sown (Targeted) in the Jena Experiment or colonized the field from the regional species pool (ExWeed; not belonging to the original species pool of the Jena Experiment). Vegetation composition across the communities studied are displayed in Fig S1.

Family	Genus	*Species*	Type	Inside the VOC cage?
**Apiaceae**	*Anthriscus*	*Anthriscus sylvestris*	Target	no
**Apiaceae**	*Carum*	*Carum carvi*	Target	no
**Apiaceae**	*Daucus*	*Daucus carota*	Target	no
**Apiaceae**	*Heracleum*	*Heracleum sphondylium*	Target	yes
**Apiaceae**	*Pastinaca*	*Pastinaca sativa*	Target	no
**Apiaceae**	*Pimpinella*	*Pimpinella major*	Target	no
**Brassicaceae**	*Capsella*	*Capsella bursa-pastoris*	ExWeed	no
**Brassicaceae**	*Cardamine*	*Cardamine pratensis*	Target	no
**Campanulaceae**	*Campanula*	*Campanula patula*	Target	yes
**Caprifoliaceae**	*Knautia*	*Knautia arvensis*	Target	yes
**Caprifoliaceae**	*Valerianella*	*Valerianella carinata*	ExWeed	no
**Caprifoliaceae**	*Valerianella*	*Valerianella locusta*	ExWeed	no
**Caryophyllaceae**	*Cerastium*	*Cerastium holosteoides*	ExWeed	yes
**Caryophyllaceae**	*Stellaria*	*Stellaria media*	ExWeed	yes
**Asteraceae**	*Achillea*	*Achillea millefolium*	Target	yes
**Asteraceae**	*Bellis*	*Bellis perennis*	Target	yes
**Asteraceae**	*Centaurea*	*Centaurea jacea*	Target	no
**Asteraceae**	*Cirsium*	*Cirsium arvense*	ExWeed	no
**Asteraceae**	*Cirsium*	*Cirsium oleraceum*	Target	yes
**Asteraceae**	*Conyza*	*Conyza canadensis*	ExWeed	no
**Asteraceae**	*Crepis*	*Crepis biennis*	Target	yes
**Asteraceae**	*Erigeron*	*Erigeron annuus*	ExWeed	no
**Asteraceae**	*Scorzoneroides*	*Scorzoneroides autumnalis*	Target	no
**Asteraceae**	*Leontodon*	*Leontodon hispidus*	Target	yes
**Asteraceae**	*Leucanthemum*	*Leucanthemum vulgare*	Target	yes
**Asteraceae**	*Solidago*	*Solidago canadensis*	ExWeed	yes
**Asteraceae**	*Sonchus*	*Sonchus asper*	ExWeed	no
**Asteraceae**	*Taraxacum*	*Taraxacum officinale*	Target	yes
**Asteraceae**	*Tragopogon*	*Tragopogon pratensis*	Target	no
**Fabaceae**	*Lathyrus*	*Lathyrus pratensis*	Target	yes
**Fabaceae**	*Lotus*	*Lotus corniculatus*	Target	yes
**Fabaceae**	*Medicago*	*Medicago lupulina*	Target	yes
**Fabaceae**	*Medicago*	*Medicago* x *varia*	Target	yes
**Fabaceae**	*Onobrychis*	*Onobrychis viciifolia*	Target	no
**Fabaceae**	*Trifolium*	*Trifolium campestre*	Target	no
**Fabaceae**	*Trifolium*	*Trifolium dubium*	Target	yes
**Fabaceae**	*Trifolium*	*Trifolium fragiferum*	Target	yes
**Fabaceae**	*Trifolium*	*Trifolium hybridum*	Target	yes
**Fabaceae**	*Trifolium*	*Trifolium pratense*	Target	yes
**Fabaceae**	*Trifolium*	*Trifolium repens*	Target	yes
**Fabaceae**	*Vicia*	*Vicia angustifolia*	ExWeed	yes

Family	Genus	*Species*	Type	Inside the VOC cage?
**Fabaceae**	*Vicia*	*Vicia cracca*	Target	yes
**Fabaceae**	*Vicia*	*Vicia sepium*	ExWeed	no
**Geraniaceae**	*Geranium*	*Geranium pratense*	Target	yes
**Geraniaceae**	*Geranium*	*Geranium spec*	ExWeed	no
**Juncaceae**	*Luzula*	*Luzula campestris*	Target	yes
**Lamiaceae**	*Ajuga*	*Ajuga reptans*	Target	yes
**Lamiaceae**	*Glechoma*	*Glechoma hederacea*	Target	yes
**Lamiaceae**	*Prunella*	*Prunella vulgaris*	Target	yes
**Papaveraceae**	*Papaver*	*Papaver rhoeas*	ExWeed	yes
**Plantaginaceae**	*Plantago*	*Plantago lanceolata*	Target	yes
**Plantaginaceae**	*Plantago*	*Plantago major*	ExWeed	no
**Plantaginaceae**	*Plantago*	*Plantago media*	Target	yes
**Plantaginaceae**	*Veronica*	*Veronica arvensis*	ExWeed	yes
**Plantaginaceae**	*Veronica*	*Veronica chamaedrys*	Target	yes
**Plantaginaceae**	*Veronica*	*Veronica persica*	ExWeed	no
**Poaceae**	*Alopecurus*	*Alopecurus pratensis*	Target	yes
**Poaceae**	*Anthoxanthum*	*Anthoxanthum odoratum*	Target	yes
**Poaceae**	*Arrhenatherum*	*Arrhenatherum elatius*	Target	yes
**Poaceae**	*Helictotrichon*	*Helictotrichon pubescens*	Target	no
**Poaceae**	*Bromus*	*Bromus erectus*	Target	yes
**Poaceae**	*Bromus*	*Bromus hordeaceus*	Target	yes
**Poaceae**	*Bromus*	*Bromus sterilis*	ExWeed	no
**Poaceae**	*Calamagrostis*	*Calamagrostis epigejos*	ExWeed	yes
**Poaceae**	*Cynosurus*	*Cynosurus cristatus*	Target	no
**Poaceae**	*Dactylis*	*Dactylis glomerata*	Target	yes
**Poaceae**	*Elytrigia*	*Elytrigia repens*	ExWeed	yes
**Poaceae**	*Festuca*	*Festuca pratensis*	Target	no
**Poaceae**	*Festuca*	*Festuca rubra*	Target	no
**Poaceae**	*Holcus*	*Holcus lanatus*	Target	yes
**Poaceae**	*Phleum*	*Phleum pratense*	Target	yes
**Poaceae**	*Poa*	*Poa compressa*	ExWeed	no
**Poaceae**	*Poa*	*Poa pratensis*	Target	yes
**Poaceae**	*Poa*	*Poa trivialis*	Target	yes
**Poaceae**	*Trisetum*	*Trisetum flavescens*	Target	yes
**Polygonaceae**	*Rumex*	*Rumex acetosa*	Target	yes
**Primulaceae**	*Primula*	*Primula veris*	Target	yes
**Ranunculaceae**	*Ranunculus*	*Ranunculus acris*	Target	yes
**Ranunculaceae**	*Ranunculus*	*Ranunculus repens*	Target	yes
**Rosaceae**	*Sanguisorba*	*Sanguisorba officinalis*	Target	no
**Rubiaceae**	*Galium*	*Galium album*	Target	yes
**Sapindaceae**	*Acer*	*Acer negundo*	ExWeed	yes
**Apiaceae**	*Anthriscus*	*Anthriscus sylvestris*	Target	no
**Apiaceae**	*Carum*	*Carum carvi*	Target	no
**Apiaceae**	*Daucus*	*Daucus carota*	Target	no
**Apiaceae**	*Heracleum*	*Heracleum sphondylium*	Target	yes
**Apiaceae**	*Pastinaca*	*Pastinaca sativa*	Target	no
**Apiaceae**	*Pimpinella*	*Pimpinella major*	Target	no
**Brassicaceae**	*Capsella*	*Capsella bursa*	ExWeed	no
**Brassicaceae**	*Cardamine*	*Cardamine pratensis*	Target	no
**Campanulaceae**	*Campanula*	*Campanula patula*	Target	yes

Family	Genus	*Species*	Type	Inside the VOC cage?
**Caprifoliaceae**	*Knautia*	*Knautia arvensis*	Target	yes
**Caprifoliaceae**	*Valerianella*	*Valerianella carinata*	ExWeed	no
**Caprifoliaceae**	*Valerianella*	*Valerianella locusta*	ExWeed	no
**Caryophyllaceae**	*Cerastium*	*Cerastium holosteoides*	ExWeed	yes
**Caryophyllaceae**	*Stellaria*	*Stellaria media*	ExWeed	yes
**Asteraceae**	*Achillea*	*Achillea millefolium*	Target	yes
**Asteraceae**	*Bellis*	*Bellis perennis*	Target	yes
**Asteraceae**	*Centaurea*	*Centaurea jacea*	Target	no
**Asteraceae**	*Cirsium*	*Cirsium arvense*	ExWeed	no
**Asteraceae**	*Cirsium*	*Cirsium oleraceum*	Target	yes
**Asteraceae**	*Conyza*	*Conyza canadensis*	ExWeed	no
**Asteraceae**	*Crepis*	*Crepis biennis*	Target	yes
**Asteraceae**	*Erigeron*	*Erigeron annuus*	ExWeed	no
**Asteraceae**	*Scorzoneroides*	*Scorzoneroides autumnalis*	Target	no
**Asteraceae**	*Leontodon*	*Leontodon hispidus*	Target	yes
**Asteraceae**	*Leucanthemum*	*Leucanthemum vulgare*	Target	yes
**Asteraceae**	*Solidago*	*Solidago canadensis*	ExWeed	yes
**Asteraceae**	*Sonchus*	*Sonchus asper*	ExWeed	no
**Asteraceae**	*Taraxacum*	*Taraxacum officinale*	Target	yes
**Asteraceae**	*Tragopogon*	*Tragopogon pratensis*	Target	no
**Fabaceae**	*Lathyrus*	*Lathyrus pratensis*	Target	yes
**Fabaceae**	*Lotus*	*Lotus corniculatus*	Target	yes
**Fabaceae**	*Medicago*	*Medicago lupulina*	Target	yes
**Fabaceae**	*Medicago*	*Medicago* x *varia*	Target	yes
**Fabaceae**	*Onobrychis*	*Onobrychis viciifolia*	Target	no
**Fabaceae**	*Trifolium*	*Trifolium campestre*	Target	no
**Fabaceae**	*Trifolium*	*Trifolium dubium*	Target	yes
**Fabaceae**	*Trifolium*	*Trifolium fragiferum*	Target	yes
**Fabaceae**	*Trifolium*	*Trifolium hybridum*	Target	yes
**Fabaceae**	*Trifolium*	*Trifolium pratense*	Target	yes
**Fabaceae**	*Trifolium*	*Trifolium repens*	Target	yes
**Fabaceae**	*Vicia*	*Vicia angustifolia*	ExWeed	yes
**Fabaceae**	*Vicia*	*Vicia cracca*	Target	yes
**Fabaceae**	*Vicia*	*Vicia sepium*	ExWeed	no
**Geraniaceae**	*Geranium*	*Geranium pratense*	Target	yes
**Geraniaceae**	*Geranium*	*Geranium spec*	ExWeed	no
**Juncaceae**	*Luzula*	*Luzula campestris*	Target	yes
**Lamiaceae**	*Ajuga*	*Ajuga reptans*	Target	yes
**Lamiaceae**	*Glechoma*	*Glechoma hederacea*	Target	yes
**Lamiaceae**	*Prunella*	*Prunella vulgaris*	Target	yes
**Papaveraceae**	*Papaver*	*Papaver rhoeas*	ExWeed	yes
**Plantaginaceae**	*Plantago*	*Plantago lanceolata*	Target	yes
**Plantaginaceae**	*Plantago*	*Plantago major*	ExWeed	no
**Plantaginaceae**	*Plantago*	*Plantago media*	Target	yes
**Plantaginaceae**	*Veronica*	*Veronica arvensis*	ExWeed	yes
**Plantaginaceae**	*Veronica*	*Veronica chamaedrys*	Target	yes
**Plantaginaceae**	*Veronica*	*Veronica persica*	ExWeed	no
**Poaceae**	*Alopecurus*	*Alopecurus pratensis*	Target	yes
**Poaceae**	*Anthoxanthum*	*Anthoxanthum odoratum*	Target	yes
**Poaceae**	*Arrhenatherum*	*Arrhenatherum elatius*	Target	yes

**Family**	**Genus**	***Species***	**Type**	**Inside the VOC cage?**
**Poaceae**	*Helictotrichon*	*Helictotrichon pubescens*	Target	no
**Poaceae**	*Bromus*	*Bromus erectus*	Target	yes
**Poaceae**	*Bromus*	*Bromus hordeaceus*	Target	yes
**Poaceae**	*Bromus*	*Bromus sterilis*	ExWeed	no
**Poaceae**	*Calamagrostis*	*Calamagrostis epigejos*	ExWeed	yes
**Poaceae**	*Cynosurus*	*Cynosurus cristatus*	Target	no
**Poaceae**	*Dactylis*	*Dactylis glomerata*	Target	yes
**Poaceae**	*Elytrigia*	*Elytrigia repens*	ExWeed	yes
**Poaceae**	*Festuca*	*Festuca pratensis*	Target	no
**Poaceae**	*Festuca*	*Festuca rubra*	Target	no
**Poaceae**	*Holcus*	*Holcus lanatus*	Target	yes
**Poaceae**	*Phleum*	*Phleum pratense*	Target	yes
**Poaceae**	*Poa*	*Poa compressa*	ExWeed	no
**Poaceae**	*Poa*	*Poa pratensis*	Target	yes
**Poaceae**	*Poa*	*Poa trivialis*	Target	yes
**Poaceae**	*Trisetum*	*Trisetum flavescens*	Target	yes
**Polygonaceae**	*Rumex*	*Rumex acetosa*	Target	yes
**Primulaceae**	*Primula*	*Primula veris*	Target	yes
**Ranunculaceae**	*Ranunculus*	*Ranunculus acris*	Target	yes
**Ranunculaceae**	*Ranunculus*	*Ranunculus repens*	Target	yes
**Rosaceae**	*Sanguisorba*	*Sanguisorba officinalis*	Target	no
**Rubiaceae**	*Galium*	*Galium album*	Target	yes

Table S2. List of volatile compounds identified at plant community level and species level. In total 127 VOCs were identified that fall into eight main chemical classes. Volatile collection was performed at community level and species level using a push pull system. Compressed air entered the system after passing through an activated charcoal filter and it was pumped out at the top through a Poropak-Q absorbent filter. After VOC collection, the traps were eluted with 200 µl dichloromethane containing nonyl acetate as an internal standard (SigmaAldrich, 10 ng $µl^{-1}$). VOCs were analyzed using a GC-MS chromatograph with helium as the carrier gas for identification and GC-FID for the quantification. Table shows the compound name with their SMILES (Simplified Molecular Input Line Entry System) notation, and the mean retention time in GC-FID and GC-MS.

ID	compound	smiles	RT FID	RT MS	*P. lanceolata*?
**Nitrogen containing compounds**					
1	benzylamine	C1=CC=C(C=C1)CN	10.67	10.30	No
2	benzyl isocyanide	[C-]#[N+]CC1=CC=CC=C1	13.50	13.15	No
3	benzothiazole	C1=CC=C2C(=C1)N=CS2	14.49	14.21	Yes
**Aromatics**					
4	p-xylene	CC1=CC=C(C=C1)C	5.99	5.41	No
5	o-xylene	CC1=CC=CC=C1C	6.26	5.99	No
6	benzaldehyde	C1=CC=C(C=C1)C=O	8.12	7.66	No
7	pseudocumene	CC1=CC(=C(C=C1)C)C	8.96	8.84	No
8	benzeneacetaldehyde	C1=CC=C(C=C1)CC=O	10.10	9.71	No
9	1,2-diethylbenzene	CCC1=CC=CC=C1CC	10.50	9.96	No
10	acetophenone	CC(=O)C1=CC=CC=C1	10.80	10.31	No
11	methylbenzoate	COC(=O)C1=CC=CC=C1	11.40	10.95	No
12	3-ethylbenzaldehyde	CCC1=CC(=CC=C1)C=O	13.25	12.73	Yes
13	4-ethylbenzaldehyde	CC(=O)C1=CC=CC=C1	13.49	13.07	Yes
14	methyl salicylate	COC(=O)C1=CC=CC=C1O	13.88	13.51	No
15	(*Z*)-3-hexenyl benzoate	CC/C=C\CCOC(=O)C1=CC=CC=C1	21.91	21.70	No
**Fatty acids derivatives**					
**Green leaf volatiles**					
16	2-hexanol	CCCCC(C)O	4.62	4.15	Yes
17	(*Z*)-3-hexenol	CC/C=C\CCO	5.73	5.17	Yes
18	(*E*)-3-hexenol	CCC=CCCO	5.85	5.24	No
19	(*Z*)-3-hexenyl acetate	CC/C=C/CCOC(=O)C	9.32	8.84	No
20	(*E*)-3-hexenyl acetate	CCC=CCCOC(=O)C	9.48	8.84	Yes
21	(*Z*)-2-hexenyl acetate	CCC/C=C/COC(=O)C	9.54	9.07	Yes
22	(*E*)-2-hexenyl acetate	CCC/C=C/COC(=O)C	9.56	9.07	Yes
23	(*Z*)-jasmone	CC/C=C\CC1=C(CCC1=O)C	18.57	18.20	No
**Other fatty acids derivatives**					
24	2-heptanone	CCCCCC(C)=O	6.53	5.95	No
25	nonane	CCCCCCCCC	6.65	6.08	No
26	heptanal	CCCCCCC=O	6.76	6.17	No
27	(*Z*)-3-hexenyl formate	CC/C=C\CCOC=O	7.20	6.64	No
28	1-octen-3-ol	CCCCCCC(C)O	8.54	8.13	Yes
29	3-octanone	CCCCCC(=O)CC	8.62	8.32	No
30	decane	CCCCCCCCCC	9.09	8.59	No
31	octanal	CCCCCCCC=O	9.23	8.70	Yes
32	(*E*)-2-undecene	CCCCCCCC/C=C/C	11.41	11.05	No
33	undecane	CCCCCCCCCCC	11.55	11.18	No
34	nonanal	CCCCCCCCCC=O	11.70	11.27	No
35	2-6-dimethyldecane	CCCCC(C)CCCC(C)C	11.72	11.34	Yes
36	isoamyl acetate	CC(C)CCOC(=O)C	12.78	12.35	No
37	3-methylheptyl acetate	CCCCC(C)CCOC(=O)C	12.80	12.46	No
38	5-methylundecane	CCCCCCC(C)CCCC	12.92	12.52	No
39	3-methylundecane	CCCCCCCCC(C)CC	13.28	12.88	No
40	(*E*)-2-nonen-1-ol	CCCCCC/C=C/CO	13.59	13.20	No
41	(*E*)-3-hexenylbutyrate	CCCC(=O)OCC/C=C/CC	13.68	13.29	No
42	decanal	CCCCCCCCCC=O	14.12	13.75	Yes
43	tetradecane	CCCCCCCCCCCCCC	18.39	18.13	No
44	decylacetate	CCCCCCCCCCOC(=O)C	18.63	18.36	No
45	pentadecane	CCCCCCCCCCCCCCC	20.45	20.22	No
46	11-tridecyn-1-ol	CC#CCCCCCCCCCO	21.26	21.01	No
47	hexadecane	CCCCCCCCCCCCCCCC	22.39	22.19	No
48	laurylacetate	CCCCCCCCCCCCOC(=O)C	22.58	22.39	No
49	heptadecane	CCCCCCCCCCCCCCCCC	24.24	24.07	No
**Terpenes**					
**Homoterpenes**					
50	(*E*)-DMNT	CC(=CCC/C(=C/C=C)/C)C	11.96	11.57	Yes
51	(*E-E*)-TMTT	CC(=CCCC(=CCCC(=CC=C)C)C)C	22.02	21.84	No
**Monoterpenes**					

ID	compound	smiles	RT FID	RT MS	*P. lanceolata*?
52	tricyclene	CC1(C2CC3C1(C3C2)C)C	7.19	6.57	No
53	α-thujene	CC1=CCC2(C1C2)C(C)C	7.34	6.77	No
54	α-pinene	CC1=CCC2CC1C2(C)C	7.41	6.92	Yes
55	β-thujene	CC1C=CC2(C1C2)C(C)C	8.33	7.94	No
56	sabinene	CC(C)C12CCC(=C)C1C2	8.45	7.99	Yes
57	β-pinene	CC1(C2CCC(=C)C1C2)C	8.52	8.03	Yes
58	β-myrcene	CC(=CCCC(=C)C=C)C	8.87	8.39	Yes
59	p-cymene	CC1=CC=C(C=C1)C(C)C	9.66	9.23	No
60	limonene	CC1=CCC(CC1)C(=C)C	9.87	9.33	Yes
61	1-8-cineole	CC1(C2CCC(O1)(CC2)C)C	9.95	9.41	No
62	(*Z*)-β-ocimene	CC(=CCC=C(C)C=C)C	10.01	9.58	Yes
63	(*E*)-β-ocimene	CC(=CC/C=C(\C)/C=C)C	10.22	9.85	Yes
64	γ-terpinene	CC1CC=C(C=C1)C(C)C	10.63	10.11	No
65	(*E*)-sabinene hydrate	CC(C)C12CCC(C1C2)(C)OC(=O)C	10.87	10.34	No
66	linalool	CC(=CCCC(C)(C=C)O)C	11.10	10.75	No
67	terpinolene	CC1=CCC(=C(C)C)CC1	11.36	10.86	No
68	(*E*)-limonene oxide	CC(=C)[C@@H]1CC[C@@]2(C(C1)O2)C	12.26	12.01	No
69	chrysanthenone	CC1=CCC2C(=O)C1C2(C)C	12.28	11.91	No
70	(*E*)-ecosmene	CC(=C)C=CC=C(C)C=C	12.59	11.97	No
71	(*Z*)-limoneneoxide	CC(=C)[C@@H]1CC[C@]2(C(C1)O2)C	12.60	12.01	No
72	nopinone	CC1(C2CCC(=O)C1C2)C	12.62	12.12	No
73	ocimene oxide	C/C(=C\CC1C(O1)(C)C)/C=C	12.73	12.28	No
74	camphor	CC1(C2CCC1(C(=O)C2)C)C	12.93	12.30	No
75	terpinene-4-ol	CC1=CCC(CC1)(C(C)C)O	13.49	13.09	No
76	2-methylisoborneol	CC1(C2CCC1(C(C2)(C)O)C)C	13.62	13.23	No
77	α-terpineol	CC1=CCC(CC1)C(C)(C)O	13.79	13.42	No
78	(*E*)-carveol	CC1=CCC(CC1O)C(=C)C	14.21	13.80	No
79	verbenone	CC1=CC(=O)C2CC1C2(C)C	14.33	13.88	No
**Sesquiterpenes**					
80	sesquiterpene1	CC1C2CCC3(C=CC(=O)C(=C3C2OC1=O)C)C	17.02	16.91	No
81	sesquiterpene2	CC1C2CCC3(C=CC(=O)C(=C3C2OC1=O)C)C	17.18	16.95	No
82	sesquiterpene3	CC1C2CCC3(C=CC(=O)C(=C3C2OC1=O)C)C	17.23	19.99	No
83	sesquiterpene4	CC1C2CCC3(C=CC(=O)C(=C3C2OC1=O)C)C	17.26	17.05	No
84	sesquiterpene5	CC1C2CCC3(C=CC(=O)C(=C3C2OC1=O)C)C	17.42	17.31	No
85	sesquiterpene6	CC1C2CCC3(C=CC(=O)C(=C3C2OC1=O)C)C	17.76	17.53	Yes
86	β-ylangene	CC(C)C1CCC2(C3C1C2C(=C)CC3)C	17.87	17.60	No
87	α-copaene	CC1=CCC2C3C1C2(CCC3C(C)C)C	17.99	17.71	Yes
88	β-bourbonene	CC1=CCC2C1C3C2(CCC3C(C)C)C	18.21	17.91	No
89	β-elemene	CC(=C)[C@@H]1CC[C@@]([C@@H](C1)C(=C)C)(C)C=C	18.34	18.05	Yes
90	patachule	CC(=CCC/C(=C/CCC(C)(C=C)O)/C)C	18.73	18.35	No
91	α-gurjunene	C[C@@H]1CC[C@@H]2[C@@H](C2(C)C)C3=C(CC[C@H]13)C	18.78	18.38	No
92	(*E*)-β-caryophyllene	C/C/1=C\CCC(=C)[C@H]2CC([C@@H]2CC1)(C)C	18.91	18.67	Yes
93	β-copaene	CC(C)[C@@H]1CC[C@]2([C@H]3C1C2CCC3=C)C	19.13	18.68	No
94	(*E*)-α-bergamotene	CC1=CCC2CC1C2(C)CCC=C(C)C	19.19	18.97	Yes
95	(*Z*)-α-bergamotene	CC1=CC[C@H]2C[C@@H]1[C@]2(C)CCC=C(C)C	19.19	18.97	No
96	sesquisabinene	CC(CCC=C(C)C)C12CCC(=C)C1C2	19.32	19.12	Yes
97	(*Z*)-β-farnesene	CC(=CCC/C(=C\CCC(=C)C=C)/C)C	19.56	19.29	No
98	(*E-E*)-α-farnesene	C(=C/C/C=C(/CC/C=C(\C)C)C)(\C=C)C	19.60	19.38	No
99	alloaromadendrene	C[C@@H]1CC[C@H]2[C@@H]1[C@H]3[C@H](C3(C)C)CCC2=C	19.70	19.49	No
100	β-selinene	CC(=C)C1CCC2(CCCC(=C)C2C1)C	19.93	19.76	No
101	(*E*)-sesquisabinene hydrate	CC(CCC=C(C)C)C12CCC(C1C2)(C)O	19.95	19.40	No
102	α-humulene	C/C/1=C\CC(/C=C/C/C(=C/CC1)/C)(C)C	19.96	19.80	No
103	γ-curcumene	CC1=CCCC(=CCC(=C(C)C)CC1)C	20.07	19.90	No
104	γ-muurolene	CC1=C[C@@H]2[C@H](CC1)C(=C)CC[C@H]2C(C)C	20.15	19.92	No
105	germacrene D	C/C/1=C\CCC(=C)/C=C/[C@@H](CC1)C(C)C	20.22	19.96	Yes
106	aristolochene	CC1CCCC2=CCC3C(C12C)C3(C)C	20.35	20.06	No
107	γ-elemene	CC(=C1CC[C@@]([C@@H](C1)C(=C)C)(C)C=C)C	20.35	20.00	No
108	eremophilene	CC1=CC2C(CC1)C(=C)CCC2C(C)C	20.50	19.59	No
109	α-muurolene	CC1=CC2C(CC1)C(=CCC2C(C)C)C	20.64	20.33	Yes
110	(*Z-E*)-α-farnesene	CC(=CCCC(=CCC=C(C)C=C)C)C	20.65	20.43	No
111	α-farsenase	CC1=CCC2C3C1C2(CCC3C(C)C)C	20.69	20.45	No
112	γ-cadinene	CC1=CCC(C=C1)[C@@H](C)CCC=C(C)C	20.85	20.62	No
113	β-cadinene	CC1=CC[C@@H]2[C@@H](C1)[C@@H](CC=C2C)C(C)C	20.98	20.78	No
114	7-epi-α-selinene	CC1=CCCC2(C1CC(CC2)C(=C)C)C	21.00	20.69	No
115	β-sesquiphellandrene	CC(CCC=C(C)C)C1CCC(=C)C=C1	21.01	20.78	No
116	sesquiterpene7	CC1C2CCC3(C=CC(=O)C(=C3C2OC1=O)C)C	21.01	20.79	Yes
117	*δ*-cadinene	CC1=CC2C(CCC(=C2CC1)C)C(C)C	21.04	20.79	No
118	(*E*)-muurolene	CC(C)C1CCC(=C)C2C1CC(=C)CC2	21.10	20.81	No
119	nerolidol	CC1CCC23C1CC(C2(C)C)CCC3=C	21.70	21.84	No
120	caryophyllene oxide 1	CC1(CC2C1CCC3(C(O3)CCC2=C)C)C	22.22	22.01	No
**Unknown**					
121	unknown_1		6.06	5.69	No
122	unknown_2		7.39	6.84	No
123	unkonwn_3		9.50	8.96	No
124	unknown_4		9.98	9.49	No
125	unknown_5		10.97	10.50	No
126	unknown_6		13.33	12.95	No
127	unknown_7		14.35	14.00	No

Table S3. Community level: Wald-chi-squared analysis of variance results for the linear mixed-effects models testing effects of plant diversity on VOC emission and diversity at community level. The effects of proportion of grass biomass, sown plant species richness, realized species diversity (Hill q1), realized phylogenetic plant richness (Hill q0) and diversity (Hill q1 and 2) were tested separately using mixed-effects models including total biomass (g FW) as covariable and fitted at the beginning of the model. All models used plot as random effects. The table reports marginal and conditional R^2 (marginal before slash and conditional after), Chi-square (X^2) and p-values for fixed effects (column), with significant effects in bold (p < 0.05) and tendencies within brackets (p < 0.1). Arrows next to the x^2 values indicate the patterns: increase (↑) or decrease (↓) in relation to the fixed factor. Data were transformed as necessary to meet assumptions and are represented as subscript numbers next to x^2-values (1 = sqrt, 2 = log10, 3 = log1p, 4 = arcsine, 5 = glmer). Sample size = 20.

Emission and diversity of VOC	Plant biomass g FW		Proportion of grass biomass g FW			Sown species diversity			Species diversity Hill q2			Phylogenetic richness Hill q0			Phylogenetic diversity Hill q1			Phylogenetic diversity Hill q2		
	x^2	*p*	$R^2_{m/c}$	x^2	*p*	$R^2_{m/c}$	x^2	*p*	$R^2_{m/c}$	x^2	*p*	$R^2_{m/c}$	x^2	*p*	$R^2_{m/c}$	x^2	*p*	$R^2_{m/c}$	x^2	*p*
**Emission**																				
Nitrogen containing	**$6.14\downarrow_5$**	**0.013**	0.02/0.70	0.31	0.575	0.01/0.45	2.35_5	0.125	0.00/0.45	2.25_5	0.133	0.01/0.62	1.32_5	0.250	0.00/0.46	2.12_5	0.145	0.00/0.44	2.56_5	0.110
Aromatics	1.29_2	0.256	0.24/0.37	**$3.88\uparrow_2$**	**0.049**	0.31/0.33	**$5.89\uparrow_2$**	**0.015**	0.07/0.08	0.03_2	0.861	0.07/0.08	0.05_2	0.822	0.07/0.08	0.01_2	0.935	0.07/0.09	0.04_2	0.837
Green leaf volatiles	0.09_1	0.762	0.05/0.44	1.36	0.244	0.19/0.75	**8.29↑**	**0.004**	0.09/0.63	*3.01_1*	*0.083*	0.04/0.51	1.15_1	0.284	0.05/0.55	1.57_1	0.210	0.06/0.6	1.94_1	0.164
Other fatty acids deriv.	0.07_2	0.785	0.70/0.70	**22.97↑**	**< 0.001**	0.32/0.32	**$7.26\uparrow_2$**	**0.007**	0.00/0.00	0.00_2	0.989	0.03/0.03	0.49_2	0.482	0.01/0.01	0.09_2	0.768	0.02/0.02	0.39_2	0.533
Homoterpenes	**$4.85\uparrow_2$**	**0.028**	0.29/0.29	2.22	0.136	0.22/0.28	0.02_2	0.876	0.22/0.28	0.06_2	0.810	0.22/0.28	0.00_2	0.963	0.23/0.27	0.04_2	0.841	0.23/0.27	0.04_2	0.852
Monoterpenes	**$9.38\uparrow_2$**	**0.002**	0.40/0.40	0.00	0.979	0.50/0.50	**$4.10\uparrow_2$**	**0.043**	0.39/0.39	0.14_2	0.710	0.43/0.43	1.28_2	0.258	0.39/0.39	0.25_2	0.620	0.39/0.39	0.12_2	0.725
Sesquiterpenes	**$7.91\uparrow_2$**	**0.005**	0.54/0.54	**4.89↑**	**0.027**	0.49/0.5	**$4.93\uparrow_2$**	**0.026**	0.35/0.35	0.25_2	0.621	0.34/0.34	0.08_2	0.777	0.35/0.35	0.49_2	0.483	0.38/0.38	1.16_2	0.282
Total	*2.81_1*	*0.094*	0.53/0.60	**12.28↑**	**< 0.001**	0.36/0.63	**7.50↑**	**0.006**	0.15/0.15	0.15_1	0.700	0.18/0.18	0.80_1	0.372	0.15/0.15	0.17_1	0.680	0.15/0.15	0.29_1	0.592
**Diversity**																				
Richness (Hill q0)	**4.43↑**	**0.035**	0.00/00	2.92	0.087	0.35/0.47	**5.26↑**	**0.022**	0.00/00	0.27	0.603	0.00/00	0.45	0.500	0.00/00	1.05	0.305	0.00/00	1.51	0.219
Shannon (Hill q1)	0.53	0.468	0.28/0.64	**$8.80\downarrow_2$**	**0.003**	0.05/0.51	0.62	0.432	0.02/0.43	0.02	0.887	0.02/0.43	0.00	0.993	0.03/0.43	0.42	0.519	0.03/0.41	0.23	0.635
Simpson (Hill q2)	0.32_1	0.571	0.14/0.63	**$4.98\downarrow_1$**	**0.026**	0.05/0.63	1.40_1	0.236	0.01/0.51	0.02_1	0.889	0.01/0.50	0.01_1	0.941	0.01/0.49	0.08_1	0.782	0.01/0.51	0.00_1	0.969

Table S4: Community level: Wald-chi-squared analysis of variance results for the linear mixed models testing effects of plant diversity on each VOC emitted at community level. The effects of sown plant species richness, realized species diversity (hill q1), realized phylogenetic plant richness (Hill q0) and diversity (Hill q1 and 2) were tested separately using mixed-effects models including fresh weight biomass as covariable and fitted at the beginning of the model. All models used plot as random effects. The table reports marginal and conditional R^2 (marginal before slash and conditional after), Chi-square (X^2) and p-values for fixed effects (column), with significant effects in bold (FDR-adjusted $p < 0.05$) and tendencies within brackets (FDR-adjusted $p < 0.1$). Arrows next to the x^2 values indicate the patterns: increase (↑) or decrease (↓) in relation to the fixed factor. Data were transformed as necessary to meet assumptions and are represented as subscript numbers next to x^2 values (1 = sqrt, 2 = log10, 3 = log1p, 4 = arcsine, 5 = glmer). Sample size = 20.

ID	Compound	Total biomass (g FW)		Grass biomass proportion		Sown plant richness		Species diversity (Hill q1)		Phylogenetic richness (Hill q0)		Phylogenetic diversity (Hill q1)	
		x^2	*p*	x^2	*p*	x^2	*p*	x^2	*p*	x^2	*p*	x^2	*p*
**Nitrogen containing compounds**													
1	benzylamine	3.35	0.134	0.41	0.523	0.57	0.449	0.07	0.796	0.13	0.722	0.05	0.827
2	benzyl isocyanide	0.24	0.627	10.78↑	**0.002**	1.56	0.423	0.06	0.806	2.34	0.253	1.94	0.327
3	benzothiazole	0.18	0.673	0.26	0.673	1.03	0.619	0.19	0.673	3.72	0.108	3.21	0.147
**Aromatics**													
4	p-xylene	0.33	0.897	0.02	0.897	0.04	0.845	0.02	0.876	0.04	0.846	0.15	0.699
5	o-xylene	0.28	0.598	0.63	0.598	0.05	0.824	1.53	0.431	0.15	0.697	0.34	0.598
6	benzaldehyde	0.22	0.638	2.26	0.265	0.01	0.924	0.20	0.655	2.61	0.212	0.93	0.638
7	pseudocumene	0.16	0.807	0.06	0.807	4.91	0.053	**6.5↓**	**0.022**	**6.88↓**	**0.017**	**7.66↓**	**0.011**
8	benzeneacetaldehyde	1.41	0.469	0.26	0.608	0.29	0.590	1.79	0.234	2.86	0.182	0.24	0.623
9	1,2-diethylbenzene	0.20	0.652	**15.87↑**	**<0.001**	**5.45↑**	**0.039**	0.08	0.784	0.02	0.885	0.83	0.652
10	acetophenone	0.00	0.952	0.10	0.952	2.33	0.254	0.70	0.804	3.48	0.124	2.46	0.234
11	methylbenzoate	**8.53↓**	**0.007**	0.14	0.707	0.03	0.861	0.92	0.336	3.47	0.063	2.35	0.125
12	3-ethylbenzaldehyde	0.16	0.717	0.13	0.717	0.00	0.987	2.25	0.267	0.68	0.692	0.59	0.692
13	4-ethylbenzaldehyde	0.01	0.931	2.02	0.311	**8.39↑**	**0.008**	1.60	0.411	0.36	0.931	1.88	0.340
14	methyl salicylate	**7.39↑**	**0.013**	0.24	0.623	1.88	0.170	1.77	0.184	**7.22↑**	**0.007**	**4.08↑**	**0.043**
15	(*Z*)-3-hexenyl benzoate	**10.46↑**	**0.002**	0.49	0.482	1.94	0.164	0.30	0.581	1.04	0.308	0.04	0.838
**Fatty acids derivatives**													
**Green leaf volatiles**													
16	2-hexanol	0.03	0.865	0.04	0.865	0.26	0.865	0.17	0.865	0.89	0.689	0.70	0.805
17	(*Z*)-3-hexenol	0.09	0.760	1.09	0.593	0.32	0.760	3.05	0.162	0.12	0.760	4.07	0.087
18	(*E*)-3-hexenol	2.88	0.179	0.63	0.426	0.20	0.658	1.91	0.167	0.74	0.388	1.82	0.177
19	(*Z*)-3-hexenyl acetate	0.30	0.582	**6.79↑**	**0.018**	**6.36↑**	**0.023**	0.53	0.582	0.58	0.582	0.26	0.612
20	(*E*)-3-hexenyl acetate	1.14	0.570	0.11	0.740	0.41	0.521	1.05	0.304	0.00	0.973	1.83	0.285
21	(*Z*)-2-hexenyl acetate	0.60	0.440	**6.68↑**	**0.020**	0.29	0.592	1.50	0.440	0.49	0.482	0.42	0.518
22	(*E*)-2-hexenyl acetate	0.02	0.890	0.18	0.890	0.64	0.849	0.40	0.890	0.66	0.832	0.19	0.890
23	(*Z*)-jasmone	0.08	0.773	0.72	0.773	0.00	0.962	3.01	0.166	0.60	0.773	4.30	0.076
**Other fatty acids derivatives**													
24	2-heptanone	1.03	0.311	3.00	0.167	0.28	0.595	1.42	0.311	1.48	0.311	1.58	0.311
25	nonane	**5.82↑**	**0.032**	1.34	0.247	3.82	0.051	1.39	0.238	0.70	0.403	1.16	0.281
26	heptanal	1.63	0.202	3.61	0.115	3.38	0.132	0.02	0.882	0.67	0.415	1.59	0.208
27	(*Z*)-3-hexenyl formate	1.65	0.303	1.06	0.303	1.93	0.199	1.74	0.199	2.68	0.199	0.19	0.665
28	1-octen-3-ol	0.37	0.920	0.01	0.920	**25.76↑**	**<0.001**	**6.72↑**	**0.019**	**10.58↑**	**0.002**	**5.89↑**	**0.030**
29	3-octanone	**5.48↑**	**0.025**	**5.04↑**	**0.025**	0.03	0.870	0.17	0.680	**5.86↓**	**0.019**	0.00	0.965
30	decane	1.00	0.316	**13.98↑**	**<0.001**	1.05	0.316	0.01	0.906	3.46	0.126	0.00	0.968
31	octanal	0.13	0.719	0.79	0.719	0.65	0.719	0.26	0.719	0.11	0.738	0.26	0.719
32	(*E*)-2-undecene	0.92	0.337	**19.98↑**	**<0.001**	3.48	0.125	4.66	0.062	**7.55↓**	**0.012**	4.12	0.085
33	undecane	0.96	0.326	**20.69↑**	**<0.001**	**8.24↑**	**0.008**	0.02	0.878	0.13	0.718	0.11	0.743

ID	Compound	Total biomass (g FW)		Grass biomass proportion		Sown plant richness		Species diversity (Hill q1)		Phylogenetic richness (Hill q0)		Phylogenetic diversity (Hill q1)	
		x^2	*p*	x^2	*p*	x^2	*p*	x^2	*p*	x^2	*p*	x^2	*p*
34	nonanal	0.14	0.708	2.57	0.217	4.86	0.055	0.05	0.823	0.84	0.708	0.00	0.969
35	2-6-dimethyldecane	0.15	0.701	1.10	0.587	**12.53↓**	**0.001**	**17.3↓**	**<0.001**	**11.66↓**	**0.001**	**13.79↓**	**<0.001**
36	isoamyl acetate	0.00	0.999	0.00	0.999	0.00	0.999	0.00	0.999	0.00	0.999	0.00	0.999
37	3-methylheptyl acetate	0.94	0.667	0.05	0.815	**5.6↓**	**0.036**	4.81	0.057	**5.64↓**	**0.035**	**7.06↓**	**0.016**
38	5-methylundecane	1.24	0.435	0.61	0.435	0.28	0.598	0.24	0.626	4.37	0.073	0.01	0.906
39	3-methylundecane	1.13	0.329	0.95	0.329	**5.45↑**	**0.039**	1.36	0.287	2.69	0.202	**7.87↑**	**0.010**
40	(*E*)-2-nonen-1-ol	**8.4↓**	**0.008**	0.22	0.639	1.78	0.183	0.92	0.336	0.00	0.985	1.27	0.259
41	(*E*)-3-hexenylbutyrate	0.72	0.397	1.07	0.397	0.04	0.847	4.07	0.087	0.14	0.706	**6.43↓**	**0.022**
42	decanal	0.54	0.464	**5.89↓**	**0.030**	0.58	0.464	1.64	0.402	2.98	0.168	1.76	0.369
43	tetradecane	0.02	0.936	0.01	0.936	0.19	0.896	0.44	0.896	1.68	0.391	0.49	0.896
44	decylacetate	**5.27↑**	**0.022**	**18.66↑**	**<0.001**	**8.22↑**	**0.008**	0.07	0.794	0.65	0.419	0.72	0.395
45	pentadecane	1.18	0.278	**21.03↑**	**<0.001**	**8.2↑**	**0.008**	0.01	0.917	0.41	0.522	0.35	0.555
46	11-tridecyn-1-ol	**8.58↑**	**0.007**	0.20	0.653	3.01	0.083	**7.2↑**	**0.007**	1.62	0.204	**7.96↑**	**0.005**
47	hexadecane	2.11	0.147	**6.3↑**	**0.024**	**12.59↑**	**0.001**	1.17	0.280	**7.25↑**	**0.014**	1.78	0.182
48	laurylacetate	0.88	0.348	**10.17↑**	**0.003**	**6.16↑**	**0.026**	0.05	0.824	0.29	0.591	0.06	0.800
49	heptadecane	0.00	0.998	**7.49↑**	**0.012**	2.09	0.297	0.02	0.998	0.05	0.998	0.29	0.998
**Terpenes**													
**Homoterpenes**													
50	(*E*)-DMNT	2.75	0.097	**7.08↓**	**0.016**	0.18	0.672	0.81	0.367	1.50	0.220	0.56	0.455
51	(*E-E*)-TMTT	2.13	0.145	**5.51↑**	**0.038**	**5.84↑**	**0.031**	0.03	0.857	0.15	0.698	0.03	0.859
**Monoterpenes**													
52	tricyclene	**16.96↑**	**<0.001**	0.49	0.482	0.25	0.616	1.92	0.166	0.84	0.361	3.03	0.082
53	α-thujene	**39.19↑**	**<0.001**	0.98	0.323	**6.4↑**	**0.011**	0.13	0.713	1.04	0.309	0.01	0.930
54	α-pinene	4.54	0.066	0.39	0.533	0.10	0.755	1.53	0.217	0.22	0.640	1.23	0.267
55	β-thujene	**10.61↓**	**0.002**	0.68	0.411	0.26	0.608	**4.3↑**	**0.038**	0.19	0.665	1.13	0.287
56	sabinene	3.14	0.076	4.11	0.076	2.51	0.113	1.17	0.278	0.07	0.788	0.06	0.811
57	β-pinene	**4.15↑**	**0.042**	**10.15↑**	**0.003**	**5.13↑**	**0.042**	0.24	0.621	2.28	0.131	0.02	0.893
58	β-myrcene	**8.37↑**	**0.006**	**7.69↑**	**0.006**	**4.92↑**	**0.027**	0.37	0.545	0.00	0.998	0.33	0.564
59	p-cymene	0.45	0.504	1.83	0.353	**7.35↑**	**0.013**	1.39	0.477	**5.56↑**	**0.037**	4.94	0.053
60	limonene	**15.6↑**	**0.000**	2.96	0.085	**4.36↑**	**0.037**	0.03	0.862	1.11	0.292	0.47	0.493
61	1-8-cineole	2.85	0.091	**7.56↑**	**0.012**	4.98	0.051	0.12	0.730	0.03	0.868	0.17	0.676
62	(*Z*)-β-ocimene	**9.33↑**	**0.004**	3.54	0.060	**6.37↑**	**0.012**	0.19	0.663	0.45	0.502	0.28	0.596
63	(*E*)-β-ocimene	1.57	0.420	0.50	0.478	0.22	0.642	0.97	0.324	0.24	0.621	1.27	0.259
64	γ-terpinene	3.38	0.066	3.98	0.066	0.94	0.332	0.12	0.724	1.09	0.296	0.42	0.519
65	(*E*)-sabinene hydrate	**7.97↑**	**0.010**	**5.39↓**	**0.020**	3.50	0.061	**4.48↓**	**0.034**	1.52	0.218	2.31	0.128
66	linalool	**25.12↑**	**<0.001**	0.27	0.601	3.22	0.073	1.88	0.171	**4.14↑**	**0.042**	2.51	0.113
67	terpinolene	3.13	0.077	**8.95↑**	**0.006**	4.82	0.056	4.33	0.075	0.04	0.847	**5.95↑**	**0.029**
68	(*E*)-limonene oxide	2.24	0.269	0.05	0.819	0.14	0.708	0.85	0.356	0.07	0.798	1.21	0.271
69	chrysanthenone	**5.3↑**	**0.043**	0.00	0.998	1.15	0.283	1.56	0.211	2.06	0.151	0.17	0.676
70	(*E*)-ecosmene	4.62	0.063	0.00	0.962	1.56	0.211	0.14	0.707	1.46	0.227	0.70	0.401
71	(*Z*)-limoneneoxide	**9.39↑**	**0.004**	2.36	0.124	0.08	0.783	1.32	0.250	0.18	0.668	0.76	0.383
72	nopinone	0.01	0.903	**7.1↑**	**0.015**	1.12	0.579	0.12	0.903	0.68	0.823	3.71	0.108
73	ocimene oxide	**9.43↑**	**0.004**	2.37	0.123	0.08	0.783	1.33	0.249	0.18	0.667	0.76	0.382
74	camphor	1.49	0.222	1.67	0.222	0.17	0.680	0.00	0.985	0.45	0.504	0.32	0.572
75	terpinene-4-ol	**14.82↑**	**<0.001**	3.82	0.051	0.17	0.680	2.46	0.116	0.19	0.665	1.21	0.272
76	2-methylisoborneol	3.32	0.137	0.01	0.927	3.28	0.070	0.28	0.597	**10.23↑**	**0.003**	2.52	0.112
77	α-terpineol	**9.55↑**	**0.004**	0.03	0.860	3.36	0.067	0.24	0.621	**8.65↑**	**0.003**	1.57	0.210
78	(*E*)-carveol	**7.97↑**	**0.010**	1.23	0.268	2.50	0.114	0.01	0.919	2.44	0.118	0.08	0.778
79	verbenone	**8.97↑**	**0.005**	**4.02↓**	**0.045**	1.00	0.317	0.69	0.406	1.39	0.238	0.06	0.809
**Sesquiterpenes**													
80	sesquiterpene1	0.36	0.551	**35.3↑**	**<0.001**	2.13	0.289	0.57	0.551	1.09	0.551	1.25	0.528
81	sesquiterpene2	0.44	0.505	**42.92↑**	**<0.001**	**11.31↑**	**0.002**	3.18	0.149	4.99	0.051	3.07	0.159
82	sesquiterpene3	**6.34↑**	**0.024**	**4.1↑**	**0.043**	0.23	0.634	0.00	0.976	2.15	0.143	1.59	0.207
83	sesquiterpene4	0.94	0.331	**17.58↑**	**0.000**	**10.49↑**	**0.002**	2.99	0.168	**5.25↑**	**0.044**	2.53	0.224
84	sesquiterpene5	0.07	0.789	**7.77↑**	**0.011**	2.05	0.304	0.34	0.789	1.36	0.487	0.93	0.671
85	sesquiterpene6	0.58	0.445	**14.55↑**	**0.000**	0.34	0.560	4.47	0.069	**6.28↓**	**0.024**	4.64	0.062
86	β-ylangene	0.28	0.598	**28.36↑**	**0.000**	**6.73↑**	**0.019**	1.67	0.394	2.61	0.212	1.54	0.430
87	α-copaene	0.05	0.815	0.17	0.815	0.67	0.815	0.00	0.952	1.34	0.495	0.29	0.815

ID	Compound	Total biomass (g FW)		Grass biomass proportion		Sown plant richness		Species diversity (Hill q1)		Phylogenetic richness (Hill q0)		Phylogenetic diversity (Hill q1)	
		x^2	*p*	x^2	*p*	x^2	*p*	x^2	*p*	x^2	*p*	x^2	*p*
88	β-bourbonene	**11.28↑**	**0.001**	**11.3↑**	**0.001**	**5.58↑**	**0.018**	0.28	0.595	1.59	0.207	0.54	0.464
89	β-elemene	**4.64↑**	**0.031**	**9.66↑**	**0.004**	1.29	0.255	1.44	0.230	0.02	0.882	0.65	0.421
90	patachule	**5.85↓**	**0.031**	0.11	0.746	1.44	0.230	**4.15↑**	**0.042**	0.84	0.360	1.38	0.240
91	α-gurjunene	0.09	0.759	3.40	0.130	0.77	0.759	0.14	0.759	0.25	0.759	0.62	0.759
92	(*E*)-β-caryophyllene	**12.3↑**	**0.001**	3.83	0.050	**5.73↑**	**0.017**	0.10	0.752	0.15	0.700	0.12	0.726
93	β-copaene	0.02	0.895	1.22	0.540	0.02	0.895	3.28	0.140	**5.52↓**	**0.038**	**10.06↓**	**0.003**
94	(*E*)-α-bergamotene	**14.29↑**	**<0.001**	2.26	0.133	2.21	0.137	2.05	0.153	**4.22↑**	**0.040**	**5.75↑**	**0.017**
95	(*Z*)-α-bergamotene	0.35	0.556	0.38	0.556	4.85	0.055	**5.87↑**	**0.031**	**7.56↑**	**0.012**	**9.3↑**	**0.005**
96	sesquisabinene	2.07	0.300	0.88	0.348	0.28	0.599	0.77	0.381	1.29	0.256	0.69	0.405
97	(*Z*)-β-farnesene	3.23	0.145	0.35	0.557	**9.01↑**	**0.005**	**5.44↑**	**0.039**	**12.61↑**	**0.001**	4.62	0.063
98	(*E*-*E*)-α-farnesene	0.06	0.828	0.05	0.828	0.24	0.806	**5.2↓**	**0.045**	0.59	0.806	0.52	0.806
99	alloaromadendrene	4.48	0.069	0.89	0.345	1.41	0.236	0.26	0.610	1.05	0.306	0.39	0.531
100	β-selinene	**6.45↑**	**0.011**	**16.77↑**	**<0.001**	1.07	0.300	3.24	0.072	0.14	0.704	2.46	0.117
101	(*E*)-sesquisabinene hydrate	0.63	0.852	0.00	1.000	0.48	0.488	0.14	0.705	0.00	1.000	1.93	0.328
102	α-humulene	2.76	0.193	1.11	0.291	0.80	0.372	0.46	0.498	0.20	0.654	2.41	0.121
103	γ-curcumene	**7.26↑**	**0.014**	**5.76↑**	**0.016**	**6.37↑**	**0.012**	0.49	0.485	**4.52↑**	**0.033**	0.71	0.398
104	γ-muurolene	1.26	0.261	**14.16↑**	**<0.001**	1.26	0.262	**5.63↓**	**0.035**	4.15	0.083	**7.71↓**	**0.011**
105	germacrene D	**26.08↑**	**<0.001**	**17.51↑**	**<0.001**	**4.48↑**	**0.034**	0.83	0.363	0.70	0.401	0.00	0.978
106	aristolochene	1.50	0.221	1.89	0.221	**5.28↓**	**0.043**	0.24	0.625	3.93	0.095	0.22	0.638
107	γ-elemene	**13.02↑**	**0.001**	1.97	0.161	0.09	0.769	0.17	0.678	0.93	0.334	0.00	0.974
108	eremophilene	0.24	0.627	**10.76↑**	**0.002**	1.56	0.425	0.06	0.807	2.33	0.254	1.94	0.327
109	α-muurolene	**6.73↑**	**0.009**	**22.99↑**	**<0.001**	**5.5↑**	**0.019**	1.55	0.213	0.04	0.841	1.75	0.186
110	(*Z*-*E*)-α-farnesene	0.49	0.483	**5.12↑**	**0.047**	**13.97↑**	**<0.001**	2.58	0.216	**16.53↑**	**<0.001**	**8.87↑**	**0.006**
111	α-farnesene	**26.73↑**	**0.000**	**6.18↑**	**0.013**	**5.55↑**	**0.018**	0.02	0.900	0.33	0.564	0.21	0.645
112	γ-cadinene	**4.25↑**	**0.039**	**7.09↑**	**0.016**	**4.17↑**	**0.041**	2.42	0.120	0.03	0.859	0.76	0.382
113	β-cadinene	0.74	0.459	0.55	0.459	0.01	0.943	2.77	0.192	0.33	0.567	2.18	0.279
114	7-epi-α-selinene	**18.49↑**	**<0.001**	0.01	0.920	3.43	0.064	2.81	0.093	3.57	0.059	3.29	0.070
115	β-sesquiphellandrene	0.31	0.577	1.41	0.469	4.12	0.085	0.05	0.821	1.69	0.388	0.07	0.797
116	sesquiterpene7	0.45	0.500	**40.95↑**	**<0.001**	**7.74↑**	**0.011**	2.40	0.243	3.57	0.118	1.17	0.500
117	*δ*-cadinene	2.31	0.129	**16.62↑**	**<0.001**	**5.14↑**	**0.047**	0.47	0.493	0.04	0.844	0.18	0.673
118	(*E*)-muurolene	4.56	0.065	1.10	0.294	**4.54↑**	**0.033**	**6.92↑**	**0.017**	2.82	0.093	**5.85↑**	**0.031**
119	nerolidol	**5.38↑**	**0.020**	**5.85↑**	**0.020**	**17.05↑**	**<0.001**	**7.83↑**	**0.010**	**5.46↑**	**0.020**	**6.7↑**	**0.019**
120	caryophyllene oxide 1	2.87	0.091	**7.82↑**	**0.010**	**6.68↑**	**0.019**	0.79	0.375	0.41	0.525	1.63	0.202
**Unknown**													
121	unknown_1	0.09	0.770	3.30	0.139	1.58	0.417	1.89	0.338	0.25	0.770	1.05	0.611
122	unknown_2	**5.97↑**	**0.029**	0.84	0.359	0.44	0.506	0.24	0.624	0.08	0.779	0.44	0.507
123	unkonwn_3	**9.64↑**	**0.004**	3.48	0.062	**10.02↑**	**0.002**	**6.83↑**	**0.009**	**4.72↑**	**0.030**	**8.54↑**	**0.003**
124	unknown_4	0.77	0.427	0.63	0.427	1.29	0.380	0.31	0.578	2.46	0.233	0.93	0.380
125	unknown_5	**7.43↑**	**0.013**	1.88	0.171	**8.52↑**	**0.006**	**13.24↑**	**0.001**	**5.49↑**	**0.019**	**9.92↑**	**0.003**
126	unknown_6	0.00	0.967	0.74	0.777	**8↑**	**0.009**	**7.32↑**	**0.014**	4.17	0.082	**8.01↑**	**0.009**
127	unknown_7	0.02	0.892	0.18	0.892	0.52	0.892	0.85	0.712	0.01	0.936	0.42	0.892

Table S5: Community level: Path coefficient for the fitted final structural equation model to investigate the direct and indirect effects of plant species richness on plant volatile profiles in an experimental grassland field. All estimates are standardized path coefficient from the SEM. Single headed arrows → indicate directional relationships between variables, double headed arrows ↔ indicate covariances between variables. Direct effects correspond to the individual paths (e.g., Sown richness → VOC richness) and indirect effects are the multiplied paths (e.g. [Sown richness → Soil pathogen diversity] × [Soil pathogen diversity → VOC richness]). Sample size = 20 communities. All predictors were scaled. See Fig. S6 and Fig. 4 for more details.

**Path**	**Estimate**	**Std. Estimate**	**Std. Error**	**P-value**
AMF diversity → VOC richness	0.03	-	0.04	0.447
Grass biomass → VOC emission	16.19	0.42	6.53	**0.031**
Grass biomass → LAI	-4.52	-0.12	8.23	0.591
LAI → VOC richness	-0.13	-	0.05	**0.018**
LAI → AMF diversity	1.15	1.15	0.47	**0.028**
LAI → Herbivore leaf damage	-0.07	-1.08	0.02	**0.003**
Sown richness → VOC emission	0.68	0.68	0.20	**0.005**
Sown richness → VOC richness	0.29	-	0.07	**<0.001**
Sown richness → Grass biomass	0.02	0.67	0.00	**0.002**
Sown richness → LAI	0.84	0.84	0.21	**0.001**
Sown richness → Plant phylogenetic diversity	0.64	0.64	0.19	**0.003**
Sown richness → Soil temperature	-0.89	-0.89	0.23	**0.002**
Sown richness → Soil pathogen diversity	-0.82	-0.82	0.37	**0.043**
Sown richness → AMF diversity	-0.36	-0.36	0.32	0.282
Sown richness → Herbivore leaf damage	0.04	0.65	0.02	**0.042**
Soil pathogen diversity → VOC emission	0.15	0.15	0.15	0.337
Soil pathogen diversity → VOC richness	0.08	-	0.04	**0.046**
Soil pathogen diversity → Herbivore leaf damage	0.02	0.29	0.01	0.166
Herbivore leaf damage → AMF diversity	9.47	0.65	3.95	**0.031**
Plant phylogenetic diversity → VOC emission	-0.41	-0.41	0.20	0.068
Plant phylogenetic diversity → VOC richness	-0.08	-	0.05	0.114
Plant phylogenetic diversity → Soil temperature	0.51	0.51	0.23	**0.044**
Plant phylogenetic diversity → Soil pathogen diversity	0.82	0.82	0.31	**0.020**
Soil temperature → Soil pathogen diversity	-0.33	-0.33	0.28	0.256
Soil temperature → AMF diversity	0.30	0.30	0.23	0.213
VOC emission ↔ Herbivore leaf damage	-0.34	-0.34	-	0.082
VOC richness ↔ Herbivore leaf damage	-0.38	-0.38	-	0.061

Table S6: **Species level: Wald-chi-squared analysis of variance results for the linear mixed models testing the effects of plant diversity on *Plantago lanceolata* VOC emission and leaf damage.** The effects of sown plant species richness, realized phylogenetic plant richness and diversity were tested separately using mixed-effects models. All models used plot as random effects. The table reports marginal and conditional R^2 (marginal before slash and conditional after), Chi-square (X^2) and p-values for fixed effects (column), with significant effects in bold (p < 0.05) and tendencies within brackets (p < 0.1). Arrows next to the x^2 values indicate the patterns: increase (↑) or decrease (↓) in relation to the fixed factor. Data were transformed as necessary to meet assumptions and are represented as subscript numbers next to x^2-values (1 = sqrt, 2 = log10, 3 = log1p, 4 = arcsine, 5 = glmer). Sample size = 29.

Variable	Sown species richness			Phylogenetic richness			Phylogenetic diversity		
	**$R^2_{m/c}$**	***x^2***	***p***	**$R^2_{m/c}$**	***x^2***	***p***	**$R^2_{m/c}$**	***x^2***	***p***
**Emission**									
Aromatic	0.00/0.66	0.10_2	0.755	0.00/0.56	1.07_5	0.302	0.00/0.65	0.18_2	0.674
GLV	0.10/NA	(2.89_1)	0.089	0.04/NA	1.02_1	0.313	0.08/NA	2.36_1	0.125
Homoterpene	0.00/0.54	0.03_3	0.870	**0.01/0.78**	**$5.75\downarrow_5$**	**0.016**	0.00/0.53	0.02_3	0.897
Monoterpene	0.01/0.53	0.43_1	0.511	0.02/0.70	0.92_2	0.337	0.01/0.51	0.18_1	0.668
Sesquiterpene	0.00/0.58	0.94_5	0.333	0.00/0.57	0.09_5	0.763	0.07/0.34	1.77_3	0.183
Terpene	0.01/0.57	0.31_2	0.575	0.01/0.60	0.21_2	0.650	0.00/0.56	0.17_2	0.682
Nitrogen-containing	**0.08/0.78**	**$5.9\downarrow_2$**	**0.015**	0.02/0.74	1.09_2	0.296	**0.09/0.80**	**$6.32\downarrow_2$**	**0.012**
Other	0.01/0.54	0.21_2	0.643	0.01/0.59	0.31_2	0.579	0.00/0.53	0.10_2	0.747
Total emission	0.02/0.22	0.67_2	0.412	0.01/0.2	0.36_1	0.547	0.03/0.19	0.63_1	0.428
**Diversity**									
Richness	0.08/1.00	0.99	0.321	0.00/0.34	0.08_3	0.773	0.06/1.00	0.64	0.422
Shannon	0.06/0.28	1.81_1	0.179	0.04/0.18	0.98_1	0.323	0.08/0.28	2.11_1	0.146
Simpson	0.12/0.28	(3.18_3)	0.075	0.08/0.16	2.08_3	0.150	0.14/0.29	(3.51_3)	0.061
**Leaf damage**									
Herbivory	0.03/NA	0.96_4	0.328	0.00/NA	0.02_4	0.888	0.02/NA	0.67_4	0.413
Pathogen	**0.27/0.55**	**$10.1\downarrow_4$**	**0.001**	**0.24/0.39**	**$6.80\downarrow_4$**	**0.009**	**0.22/0.45**	**$6.66\downarrow_4$**	**0.010**
Total damage	**0.18/NA**	**$5.42\downarrow_4$**	**0.020**	**0.13/NA**	**$3.96\downarrow_4$**	**0.047**	**0.15/NA**	**$4.57\downarrow_4$**	**0.033**

Table S7. Species level: Wald-chi-squared analysis of variance results for the linear mixed models testing the effects of plant diversity and leaf damage on each VOC emission of *Plantago lanceolata*. The effects of plant diversity and leaf damage were tested separately using mixed-effects models. All models used plot as random effects. The table reports marginal and conditional R^2 (marginal before slash and conditional after), Chi-square (X^2) and p-values for fixed effects (column), with significant effects in bold (FDR-adjusted *p* < 0.05) and tendencies within brackets (FDR-adjusted *p* < 0.1). Arrows next to the x^2 values indicate the patterns: increase (↑) or decrease (↓) in relation to the fixed factor. Data were log10 transformed. Sample size = 29.

Compound	Sown plant species richness		Phylogenetic plant richness		Phylogenetic plant diversity		Total leaf damage		Herbivore leaf damage		Pathogen leaf damage	
	x^2	*p*	x^2	*p*	x^2	*p*	x^2	*p*	x^2	*p*	x^2	*p*
**Aromatic**												
3-ethylbenzaldehyde	0.00	0.987	0.41	0.520	0.01	0.932	0.01	0.907	0.65	0.422	1.55	0.213
4-ethylbenzaldehyde	0.65	0.421	0.00	0.992	0.24	0.623	0.80	0.372	1.87	0.171	0.02	0.875
**Nitrogen-containing**												
benzothiazole	0.91	0.341	0.04	0.837	0.83	0.364	0.63	0.427	*2.89*	*0.089*	0.55	0.457
**GLV**												
2-hexanol	0.09	0.759	0.34	0.561	0.07	0.792	1.45	0.229	0.30	0.582	2.29	0.130
(*E*)-2-hexenyl acetate	2.28	0.131	0.90	0.343	1.23	0.268	2.33	0.127	1.86	0.173	1.46	0.226
(Z)-3-hexenol	**7.36↓**	**0.007**	**4.97↓**	**0.026**	**7.06↓**	**0.008**	**7.01↑**	**0.008**	1.58	0.209	**7.28↑**	**0.007**
(*E*)-3-hexenyl acetate	2.51	0.113	1.03	0.311	2.35	0.125	0.92	0.338	1.06	0.304	0.15	0.700
(Z)-2-hexenyl acetate	**5.91↓**	**0.015**	1.43	0.232	*3.17*	*0.075*	*3.20*	*0.074*	0.76	0.383	*3.66*	*0.056*
**Monoterpene**												
α-pinene	**3.07**	**0.049**	1.06	0.303	2.05	0.152	1.11	0.292	3.69	*0.055*	0.03	0.855
β-pinene	2.62	0.105	0.21	0.650	1.73	0.188	0.00	0.967	0.10	0.755	0.18	0.673
β-myrcene	2.85	*0.091*	1.76	0.185	2.34	0.126	0.77	0.381	0.03	0.859	1.43	0.232
(*Z*)-β-ocimene	0.10	0.758	0.02	0.891	0.12	0.730	0.27	0.606	**5.2↑**	**0.023**	2.68	0.101
sabinene	0.32	0.570	0.64	0.422	1.05	0.305	0.36	0.547	0.00	0.986	1.76	0.184
(*E*)-β-ocimene	**5.00↓**	**0.025**	1.57	0.211	*3.22*	*0.073*	0.11	0.739	0.27	0.606	0.10	0.756
limonene	0.10	0.753	0.00	0.979	0.03	0.860	0.17	0.683	0.23	0.629	1.71	0.191
**Sesquiterpene**												
β-elemene	0.10	0.751	0.02	0.899	0.02	0.883	0.07	0.795	0.07	0.793	0.31	0.578
α-copaene	0.00	0.967	0.17	0.677	0.09	0.765	0.03	0.865	0.03	0.863	0.08	0.783
α-muurolene	1.40	0.237	0.03	0.857	0.59	0.443	1.98	0.160	**4.38↑**	**0.036**	0.11	0.743
sesquiterpene7	*3.69*	*0.055*	**4.15↑**	**0.042**	**4.11↑**	**0.043**	0.52	0.470	0.07	0.796	0.99	0.319
(*E*)-α-bergamotene	0.02	0.889	0.06	0.814	0.05	0.821	0.19	0.663	0.08	0.772	0.60	0.440
sesquiterpene6	1.54	0.214	0.30	0.587	1.00	0.318	1.72	0.189	**5.03↑**	**0.025**	0.00	0.945
sesquisabinene	0.64	0.423	0.75	0.385	0.34	0.561	*3.24*	*0.072*	0.24	0.627	**7.20↓**	**0.007**
germacrene d	1.38	0.240	2.49	0.115	1.65	0.198	*3.08*	*0.079*	0.03	0.859	**8.81↓**	**0.003**
β-caryophyllene	0.02	0.896	0.04	0.849	0.06	0.805	0.01	0.923	0.01	0.921	0.06	0.800
**Homoterpene**												
(E)-DMNT	0.04	0.846	0.93	0.336	0.23	0.635	0.61	0.433	0.25	0.617	2.45	0.117
**Other**												
octanal	0.01	0.931	0.10	0.754	0.00	0.978	0.30	0.582	0.00	0.967	1.04	0.309
dodecane	0.48	0.487	0.78	0.377	1.14	0.285	0.09	0.758	0.35	0.556	0.94	0.333
decanal	0.03	0.861	0.16	0.690	0.04	0.836	1.93	0.165	0.48	0.490	2.32	0.128
1-octen-3-ol	0.21	0.648	0.37	0.543	0.54	0.464	0.00	0.947	0.01	0.912	0.06	0.804

Table S8. Species level: Wald-chi-squared analysis of variance results for the linear mixed models testing the effects of Community VOC on *Plantago lanceolata* VOC emission and leaf damage. The effects community VOC profiles were tested separately using mixed-effects models. All models used plot as random effects. The table reports marginal and conditional R^2 (marginal before slash and conditional after), Chi-square (X^2) and p-values for fixed effects (column), with significant effects in bold (p < 0.05) and tendencies within brackets (p < 0.1). Arrows next to the x^2 values indicate the patterns: increase (↑) or decrease (↓) in relation to the fixed factor. Data were transformed as necessary to meet assumptions and are represented as subscript numbers next to x^2 values (1 = sqrt, 2 = log10, 3 = log1p, 4 = arcsine, 5 = glmer). Sample size = 29.

Variable	Community Green leaf volatiles			Community VOC Monoterpenes			Community VOC Sesquiterpenes			Community VOC richness			Community VOC Shannon diversity			Community VOC Simpson diversity		
	$R^2_{m/c}$	x^2	*p*	$R^2_{m/c}$	x^2	*p*	$R^2_{m/c}$	x^2	*p*	$R^2_{m/c}$	x^2	*p*	$R^2_{m/c}$	x^2	*p*	$R^2_{m/c}$	x^2	*p*
**Emission**																		
Aromatic	0.00/0.50	0.01_1	0.940	0.00/0.65	0.20_2	0.651	0.00/0.66	0.01_2	0.942	0.00/0.66	0.00_2	0.959	0.06/0.46	2.19_1	0.139	0.06/0.46	2.19_1	0.139
GLV	**0.14/NA**	**$4.26\downarrow_2$**	**0.039**	0.03/NA	0.97_1	0.326	0.03/0.03	0.81_1	0.369	0.11/NA	3.13_1	0.077	0.03/NA	0.85_1	0.357	0.03/NA	0.85_1	0.357
Homoterpene	0.01/0.50	0.19_3	0.662	0.01/0.53	0.30_3	0.586	0.01/0.57	0.36_3	0.551	0.00/0.54	0.01_3	0.908	**0.22/0.56**	**$9.13\uparrow_3$**	**0.003**	**0.22/0.56**	**$9.13\uparrow_3$**	**0.003**
Monoterpene	0.00/0.50	0.01_1	0.920	0.00/0.50	0.23_1	0.628	0.00/0.50	0.11_1	0.739	0.03/0.59	1.73_1	0.188	0.03/0.62	1.66_2	0.198	0.03/0.62	1.66_2	0.198
Sesquiterpene	0.02/0.42	0.45_3	0.504	0.07/0.31	2.32_3	0.127	0.04/0.26	1.23_3	0.268	0.03/0.37	0.98_3	0.323	0.04/0.24	0.91_3	0.340	0.04/0.24	0.91_3	0.340
Total terpene	0.01/0.62	0.30_2	0.586	0.03/0.38	0.98_1	0.322	0.00/0.56	0.09_2	0.764	0.01/0.60	0.71_2	0.400	0.03/0.53	1.27_2	0.260	0.03/0.53	1.27_2	0.260
Nitrogen-containing	**0.15/0.74**	**$8.4\downarrow_2$**	**0.004**	**0.11/0.83**	**$11.59\downarrow_2$**	**0.001**	**0.04/0.78**	**$4.29\downarrow_2$**	**0.038**	**0.06/0.75**	**$4.95\downarrow_2$**	**0.026**	0.00/0.68	0.10_2	0.748	0.00/0.68	0.10_2	0.748
Other	0.02/0.64	0.84_2	0.360	0.01/0.52	0.69_2	0.406	0.00/0.53	0.02_2	0.895	0.00/0.55	0.12_2	0.724	0.00/0.52	0.12_2	0.731	0.00/0.52	0.12_2	0.731
Total emission	0.16/NA	2.37_2	0.124	0.00/0.19	0.09_1	0.771	0.01/0.20	0.20_1	0.654	0.04/0.16	0.98_1	0.323	0.05/0.14	1.06_1	0.304	0.05/0.14	1.06_1	0.304
**Diversity**																		
Richness	**1.00/NA**	**$6.55\downarrow$**	**0.011**	0.02/1.00	0.23	0.633	0.02/1.00	0.25	0.614	0.01/1.00	0.13	0.715	**0.65/1.00**	**$6.47\uparrow$**	**0.011**	**0.65/1.00**	**$6.47\uparrow$**	**0.011**
Shannon	0.01/0.29	0.22_1	0.636	0.11/0.27	3.68_3	0.055	0.09/0.18	2.41_1	0.121	0.05/0.30	1.57_1	0.210	0.13/NA	2.23_1	0.136	0.13/NA	2.23_1	0.136
Simpson	0.08/0.39	1.26_3	0.262	0.20/0.28	**$6.50\uparrow_3$**	**0.011**	0.17/0.18	**$4.73\uparrow_3$**	**0.030**	0.06/0.25	1.67_3	0.196	0.03/0.05	0.41_3	0.522	0.03/0.05	0.41_3	0.522
**Leaf damage**																		
Herbivory	**0.14/NA**	**$4.19\downarrow_4$**	**0.041**	0.04/NA	1.00_4	0.317	0.01/NA	0.32_4	0.572	0.05/NA	1.53_4	0.217	0.03/NA	0.78_4	0.376	0.04/NA	1.13_4	0.289
Pathogen	0.07/0.56	$2.06T_4$	0.151	**0.21/0.38**	**$7.36\downarrow_4$**	**0.007**	**0.34/0.53**	**$14.25\downarrow_4$**	**<0.001**	**0.18/0.62**	**$8.78\downarrow_4$**	**0.003**	0.05/0.44	1.98_4	0.159	0.08/0.51	3.05_4	0.081
Total damage	0.01/NA	$0.37T_4$	0.544	**0.24/NA**	**$7.72\downarrow_4$**	**0.005**	**0.24/NA**	**$7.87\downarrow_4$**	**0.005**	0.09/NA	2.57_4	0.109	0.01/NA	0.36_4	0.546	0.02/NA	0.61_4	0.437

Table S9: **Species level: Wald-chi-squared analysis of variance results for the linear mixed models the effects of Community VOC on each VOC emission of *Plantago lanceolata*.** The effects community VOC profiles were tested separately using mixed-effects models. All models used plot as random effects. The table reports marginal and conditional R^2 (marginal before slash and conditional after), Chi-square (X^2) and p-values for fixed effects (column), with significant effects in bold (FDR-adjusted $p < 0.05$) and tendencies within brackets (FDR-adjusted $p < 0.1$). Arrows next to the x^2 values indicate the patterns: increase (↑) or decrease (↓) in relation to the fixed factor. Data were log10 transformed. Sample size = 29.

**Compound**	**Community Green leaf volatiles**		**Community VOC Monoterpenes**		**Community VOC Sesquiterpenes**		**Community VOC Richness**		**Community VOC Shannon**		**Community VOC Simpson**	
	x^2	***p***	***x^2***	***p***	***x^2***	***p***	***x^2***	***p***	***x^2***	***p***	***x^2***	***p***
**Aromatic**												
3-ethylbenzaldehyde	1.82	0.177	0.29	0.588	1.10	0.295	0.09	0.770	0.73	0.393	1.94	0.163
4-ethylbenzaldehyde	1.03	0.311	0.00	0.963	0.11	0.736	0.33	0.568	(3.01)	*0.083*	3.80	*0.051*
**Nitrogen**												
benzothiazole	(3.84)	*0.050*	1.14	0.285	0.17	0.680	0.29	0.590	0.98	0.321	1.41	0.236
**GLV**												
2-hexanol	3.21	*0.073*	0.69	0.405	0.96	0.326	0.01	0.933	0.58	0.446	1.32	0.251
(*E*)-2-hexenyl acetate	2.67	0.102	1.18	0.277	(3.61)↑	*0.057*	**3.97↓**	**0.046**	1.09	0.298	1.06	0.303
(Z)-3-hexenol	1.10	0.295	**5.39↓**	**0.020**	3.31	*0.069*	1.35	0.245	1.73	0.189	1.71	0.191
(*E*)-3-hexenyl acetate	**4.15↓**	**0.042**	1.29	0.257	0.78	0.378	2.16	0.142	0.60	0.438	1.22	0.268
(Z)-2-hexenyl acetate	**6.88↓**	**0.009**	**3.97↓**	**0.046**	**6.09↓**	**0.014**	**6.68↓**	**0.010**	**4.97↑**	**0.026**	**5.3↑**	**0.021**
**Monoterpene**												
α-pinene	2.94	*0.086*	0.40	0.529	0.31	0.575	0.73	0.393	**7.94↑**	**0.005**	**8.89↑**	**0.003**
β-pinene	**10.20↓**	**0.001**	0.70	0.402	0.11	0.740	2.07	0.150	**4.44↑**	**0.035**	**6.45↑**	**0.011**
β-myrcene	0.03	0.864	(3.06)	*0.080*	(3.15)	*0.076*	1.45	0.229	0.00	0.989	0.07	0.788
(*Z*)-β-ocimene	(2.88)	*0.090*	0.09	0.765	0.49	0.484	0.33	0.566	0.16	0.687	1.07	0.302
sabinene	1.20	0.273	0.65	0.421	0.07	0.797	0.16	0.689	(3.42)	*0.064*	(3.69)	*0.055*
(*E*)-β-ocimene	**6.10↓**	**0.014**	1.43	0.232	1.34	0.247	1.63	0.202	**12.7↑**	**0.000**	**14.17↑**	**0.000**
limonene	0.06	0.812	1.60	0.207	1.17	0.279	0.28	0.594	0.40	0.525	1.37	0.242
**Sesquiterpene**												
β-elemene	0.57	0.449	0.12	0.727	0.00	0.975	0.12	0.728	1.29	0.256	1.54	0.214
α-copaene	1.13	0.288	1.56	0.212	0.41	0.520	0.04	0.835	**3.87↑**	**0.049**	2.67	0.102
α-muurolene	**5.04↓**	**0.025**	(3.60)	*0.058*	2.37	0.124	1.13	0.288	**6.49↑**	**0.011**	**5.53↑**	**0.019**

**Compound**	**Community Green leaf volatilives**		**Community VOC Monoterpenes**		**Community VOC Sesquiterpenes**		**Community VOC Richness**		**Community VOC Shannon**		**Community VOC Simpson**	
	x^2	***p***	x^2	***p***	x^2	***p***	x^2	***p***	x^2	***p***	x^2	***p***
sesquiterpene7	0.07	0.789	**5.7↑**	**0.017**	**4.68↑**	**0.031**	1.10	0.295	0.02	0.895	0.15	0.695
(*E*)-α-bergamotene	1.90	0.168	1.35	0.245	0.12	0.730	0.17	0.679	**5.58↑**	**0.018**	**4.86↑**	**0.027**
sesquiterpene6	**4.81↓**	**0.028**	1.64	0.200	0.81	0.367	0.18	0.670	**11.57↑**	**0.001**	**9.81↑**	**0.002**
sesquisabinene	0.79	0.373	0.90	0.344	2.58	0.109	0.44	0.507	0.09	0.770	0.55	0.458
germacrene d	1.25	0.264	(3.08)	*0.079*	(3.40)	*0.065*	0.37	0.542	1.54	0.215	1.32	0.250
β-caryophyllene	0.12	0.725	0.00	0.979	0.54	0.463	0.74	0.391	0.28	0.595	0.20	0.656
**Homoterpene**												
(E)-DMNT	(3.10)	*0.076*	0.70	0.402	0.72	0.396	0.26	0.613	1.81	0.178	(3.17)	*0.075*
**Other**												
octanal	2.03	0.154	0.51	0.474	0.31	0.580	0.77	0.381	0.05	0.822	0.73	0.394
dodecane	0.28	0.599	1.43	0.232	0.12	0.726	0.04	0.845	1.52	0.217	2.12	0.145
decanal	0.85	0.357	0.57	0.451	0.44	0.505	0.33	0.563	0.03	0.865	0.26	0.612
1-octen-3-ol	1.68	0.195	1.97	0.161	0.29	0.592	0.00	0.948	**3.9↑**	**0.048**	**4.08↑**	**0.043**

Table S10: Species level: Wald-chi-squared analysis of variance results for the linear mixed models the effects of leaf damage on *Plantago lanceolata* VOC emission. The effects of leaf damage were tested separately using mixed-effects models. All models used plot as random effects. The table reports marginal and conditional R^2 (marginal before slash and conditional after), Chi-square (X^2) and p-values for fixed effects (column), with significant effects in bold ($p < 0.05$) and tendencies within brackets ($p < 0.1$). Arrows next to the x^2 values indicate the patterns: increase (↑) or decrease (↓) in relation to the fixed factor. Data were transformed as necessary to meet assumptions and are represented as subscript numbers next to x^2- values (1 = sqrt, 2 = log10, 3 = log1p, 4 = arcsine, 5 = glmer). Sample size = 29.

**Variable**	**Herbivore damage**			**Pathogen damage**			**Total damage**		
	**R^2**	**x^2**	***p***	**R^2**	**x^2**	***p***	**R^2**	**x^2**	***p***
**Emission**									
Aromatic	0.00/0.65	0.05_2	0.824	0.01/0.48	0.30_1	0.586	0.00/0.52	0.20_1	0.653
GLV	0.04/NA	1.19_1	0.275	0.00/NA	0.08_1	0.774	0.03/NA	0.71_1	0.398
Homoterpene	0.00/0.56	0.23_3	0.635	0.01/0.58	0.27_3	0.606	0.00/0.53	0.01_3	0.919
Monoterpene	0.01/0.50	0.50_1	0.477	0.04/0.45	1.11_1	0.292	0.00/0.51	0.00_1	0.989
Sesquiterpene	0.00/0.32	0.09_3	0.768	0.16/0.23	$(3.17_3)\downarrow$	*0.075*	0.04/0.29	1.45_3	0.228
Terpene	0.00/0.56	0.07_2	0.791	0.10/0.32	2.46_1	0.117	0.02/0.38	0.74_1	0.391
Nitrogen-containing	**0.01/0.43**	$\mathbf{10.01\uparrow_5}$	**0.002**	0.01/0.71	0.30_2	0.582	0.03/0.74	(2.96_2)	0.085
Other	0.00/0.56	0.25_2	0.615	0.02/0.48	0.58_2	0.446	0.01/0.53	0.44_2	0.507
Total	0.01/0.16	0.20_1	0.656	0.00/0.16	0.04_1	0.845	0.00/0.18	0.02_1	0.899
**Diversity**									
Richness	0.08/1.00	0.74	0.391	0.06/1.00	0.73	0.392	0.04/1.00	0.57	0.450
Shannon	0.00/0.19	0.00_1	0.982	0.16/0.18	(3.66_1)	0.056	0.03/0.18	0.92_1	0.338
Simpson	0.00/0.12	0.04_3	0.850	**0.22/NA**	$\mathbf{6.47\downarrow_1}$	**0.011**	0.07/0.14	2.12_3	0.145

References

1. W. W. Weisser et al., Biodiversity effects on ecosystem functioning in a 15-year grassland experiment: Patterns, mechanisms, and open questions. Basic and Applied Ecology 23, 1-73 (2017).

2. Y. Y. Huang et al., Enhanced stability of grassland soil temperature by plant diversity. Nature Geoscience 17 (2024).

3. S. T. Meyer et al., Consistent increase in herbivory along two experimental plant diversity gradients over multiple years. Ecosphere 8 (2017).

4. C. Albracht et al., Common soil history is more important than plant history for arbuscular mycorrhizal community assembly in an experimental grassland diversity gradient. Biology and Fertility of Soils 60, 547-562 (2024).

5. R. Dinnage, Phylogenetic diversity of plants alters the effect of species richness on invertebrate herbivory. PeerJ 1 (2013).

6. D. Francioli, J. v. Ruijven, L. Bakker, L. Mommer, Drivers of total and pathogenic soil-borne fungal communities in grassland plant species. Fungal Ecology 48 (2020).

7. B. Randlkofer, E. Obermaier, M. Hilker, T. Meiners, Vegetation complexity-The influence of plant species diversity and plant structures on plant chemical complexity and arthropods. Basic and Applied Ecology 11, 383-395 (2010).

8. R. Escobar-Bravo, P. A. Lin, J. M. Waterman, M. Erb, Dynamic environmental interactions shaped by vegetative plant volatiles. Natural Product Reports 40, 840-865 (2023).

9. N. Eisenhauer et al., The multiple-mechanisms hypothesis of biodiversity–stability relationships. Basic and Applied Ecology 79, 153-166 (2024).
